# Frequent gene flow blurred taxonomic boundaries of sections in *Lilium* L. (Liliaceae)

**DOI:** 10.1371/journal.pone.0183209

**Published:** 2017-08-25

**Authors:** Xun Gong, Kuo-Hsiang Hung, Yu-Wei Ting, Tsai-Wen Hsu, Lenka Malikova, Huyen Trang Tran, Chao-Li Huang, Shih-Hui Liu, Tzen-Yuh Chiang

**Affiliations:** 1 Key Laboratory for Plant Diversity and Biogeography of East Asia, Kunming Institute of Botany, Chinese Academy of Sciences, Kunming, China; 2 Graduate Institute of Bioresources, National Pingtung University of Science and Technology, Pingtung, Taiwan; 3 Department of Life Sciences, National Cheng Kung University, Tainan, Taiwan; 4 Endemic Species Research Institute, Nantou, Taiwan; 5 Institute of Botany CAS, Třeboň, Czech Republic; 6 Institute of Natural Science Education, Vinh University, Vinh, Nghe An, Vietnam; 7 Institute of Tropical Plant Sciences, National Cheng Kung University, Tainan, Taiwan; 8 Department of Biology, Saint Louis University, Saint Louis, Missouri, United States of America; 9 Missouri Botanical Garden, Saint Louis, Missouri, United States of America; 10 University Center for Bioscience and Biotechnology, National Cheng Kung University, Tainan, Taiwan; Università di Pisa, ITALY

## Abstract

Gene flow between species may last a long time in plants. Reticulation inevitably causes difficulties in phylogenetic reconstruction. In this study, we looked into the genetic divergence and phylogeny of 20 *Lilium* species based on multilocus analyses of 8 genes of chloroplast DNA (cpDNA), the internally transcribed nuclear ribosomal DNA (nrITS) spacer and 20 loci extracted from the expressed sequence tag (EST) libraries of *L*. *longiflorum* Thunb. and *L*. *formosanum* Wallace. The phylogeny based on the combined data of the maternally inherited cpDNA and nrITS was largely consistent with the taxonomy of *Lilium* sections. This phylogeny was deemed the hypothetical species tree and uncovered three groups, i.e., Cluster A consisting of 4 taxa from the sections *Pseudolirium* and *Liriotypus*, Cluster B consisting of the 4 taxa from the sections *Leucolirion*, *Archelirion* and *Daurolirion*, and Cluster C comprising 10 taxa mostly from the sections *Martagon* and *Sinomartagon*. In contrast, systematic inconsistency occurred across the EST loci, with up to 19 genes (95%) displaying tree topologies deviating from the hypothetical species tree. The phylogenetic incongruence was likely attributable to the frequent genetic exchanges between species/sections, as indicated by the high levels of genetic recombination and the IMa analyses with the EST loci. Nevertheless, multilocus analysis could provide complementary information among the loci on the species split and the extent of gene flow between the species. In conclusion, this study not only detected frequent gene flow among *Lilium* sections that resulted in phylogenetic incongruence but also reconstructed a hypothetical species tree that gave insights into the nature of the complex relationships among *Lilium* species.

## Introduction

Recent progress in molecular technologies has made extensive molecular data available for phylogenetic studies [1]. With advanced techniques and big data, the understanding of the evolutionary relationships between living organisms has increased dramatically. Meanwhile, new challenges in the field of phylogenetics have arisen. Phylogenetic incongruence is a ubiquitous problem in phylogenetic reconstruction [2–4]. To get insights into the incongruence, Rokas *et al*. [2,5] and Hollingsworth *et al*. [6] suggest interpreting both the analytical and biological factors involved in the phylogenetic conflict. Though each individual factor has been examined by earlier studies empirically and/or theoretically (e.g., [7–9]), studies rarely inspect both the analytical and biological factors with empirical data. In our study, the commercially and ethnobotanically important genus *Lilium* was investigated to explore how both factors were responsible for the phylogenetic incongruence.

Analytical factors include the limitations and defects of the methods, while the biological factors comprise several evolutionary forces that are involved in the phylogenetic conflict. Incomplete lineage sorting, gene flow between taxa, horizontal transfer from the organellar genome, natural selection, and polyploidization are recognized as common biological factors that contribute to phylogenetic incongruence [10–13]. Among these factors, interspecific gene flow often leads to a reticulate evolutionary history [14–15]. While the incongruences stemming from gene flow between the populations or the closely related species are broadly addressed [16–18], the incongruences rooted in the genus-wide or higher-level interspecific gene flow are not well explained [19–20]. Because loci may experience different histories with different paces, combining loci inevitably causes systematic conflicts. There has been considerable debates on the combining multilocus sequence data for phylogenetic reconstruction. Most studies suggest that multilocus analysis effectively extends the number of evolutionarily informative characters and thus increases the accuracy of the phylogeny even with incomplete taxon sampling [21–24]. However, some studies indicate that multilocus sequences also unavoidably reduce the discriminatory power of the phylogenetic analysis [2,25].

The genus *Lilium* L. (Liliaceae), true lilies, is a group of herbs that are important worldwide for medicine, food, and horticulture [26–29]. This genus occurs in Eurasia and North America and is the most abundant in the Hengduan Mountain Region (HMR) of Southwest China and the Himalayan Mountain Ranges [26,30]. China is considered to be the biodiversity center of this genus, especially Sichuan, Yunnan, and Tibet [31–32], with a total of 55 species [29]. In total, approximately 100 *Lilium* species are classified into 5 to 11 sections [30, 33–35]. Seven sections in the genus sensu Comber [30] were applied in this study. High levels of morphological variation in *Lilium* lead to difficulties in the section delimitation. Previous phylogenetic studies suggest that most of the sections are polyphyletic, and some of these studies detected systematic incongruence among the loci [36–44]. Nevertheless, further clarifications on the mechanisms resulting in this incongruence were missing in all these studies. The fundamental understanding of the phylogenetic conflicts in *Lilium* makes the genus an ideal system for testing both the biological and analytical factors that are involved in phylogenetic incongruence.

There are two primary purposes in our study: 1) using multi-locus analyses to reconstruct the phylogeny of *Lilium* and to provide phylogenetic implications on the current section delimitation [30] and 2) to test whether analytical and/or biological factors caused the phylogenetic incongruence of *Lilium*. The incongruence caused by polyploidization was not discussed because most of the *Lilium* species are diploid, except for the triploid *L*. *tigrinum* [45–46]. In this study, we discussed how incomplete lineage sorting and interspecific gene flow can result in phylogenetic conflicts.

## Materials and methods

### Sampling and DNA extraction

In total, 29 *Lilium* samples, representing 20 species in seven sections, were collected from Taiwan, France, Japan, and China (Sichuan, Shandong, Yunnan, and Hubei Provinces). We confirm that the field studies did not involve endangered or protected species. No permission was needed because the samples were not collected in a protected area. The sample information is shown in Table 1. Leaf materials were dried with silica gel and stored at -80°C for later experiments. Genomic DNA from each sample was extracted using a CTAB method [47], diluted to 2 ng/μL with TE solution, and stored at -20°C.

**Table 1:** Information of the investigated *Lilium* samples.

Species	Sections	Species codes	Number of samples	Localities and altitudes	Voucher information
*L*. *speciosum* var. *gloriosoides*	*Archelirion*	spe	1	Taiwan: Yilan (24.94N, 121.88E), 442 m	C.-C. Huang 42
*L*. *speciosum* var. *gloriosoides*	*Archelirion*	gl8	1	China: Fugong, Yunnan (26.53N, 98.89E), 2700 m	X. Gong PD008
*L*. *speciosum* var. *gloriosoides* (cultivated)	*Archelirion*	spe	1	Taiwan: Shenkeng, Taipei (24.99N, 121.61N), 480 m	Y.-W. Ting 1
*L*. *maculatum*	*Daurolirion*	mac	2	Japan: Izu, Shizuoka (34.97N, 138.95E), 44 m	N. Osada J1, J2
*L*. *formosanum*	*Leucolirion*	for	1	Taiwan: Taoyuan (24.77N, 121.35E), 1491 m	C.-C. Huang Lf51
*L*. *leucanthum*	*Leucolirion*	leu	1	China: Yichang, Hubei (30.67N, 111.25E)	X. Gong PD013
*L*. *sargentiae*	*Leucolirion*	sar	1	China: Sichuan (32.15N, 107.26E)	X. Gong PD011
*L*. *sulphureum*	*Leucolirion*	sul	1	China: Lijiang, Yunnan (26.88N,100.23E)	X. Gong PD010
*L*. *bulbiferum* (cultivated)	*Liriotypus*	bul	2	France: Alpine Botanical Garden du Lautaret (45.03N, 6.40E), 2100 m	L. Malikova PD044, PD048
*L*. *pyrenaicum* (cultivated)	*Liriotypus*	pyr	2	France: Alpine Botanical Garden du Lautaret (45.03N, 6.40E), 2100 m	L. Malikova PD038, PD039
*L*. *monadelphum* (cultivated)	*Liriotypus*	mon	2	France: Alpine Botanical Garden du Lautaret (45.03N, 6.40E), 2100 m	L. Malikova PD050, PD051
*L*. *martagon* (cultivated)	*Martagon*	mar	1	France: Alpine Botanical Garden du Lautaret (45.03N, 6.40E), 2100 m	L. Malikova PD034
*L*. *tsingtauense*	*Martagon*	tsi	2	China: Laoshan, Shandong (36.18N, 120.58E), 700 m	X. Gong PD020, PD021
*L*. *‘Casa Blanca’* (cultivated)	Not specified	cas	1	Taiwan: Taipei (25.07N, 121.53E), 170 m	Y.-W. Ting P
*L*. *parryi* (cultivated)	*Pseudolirium*	ryi	1	USA: Native Botanical Garden of Southern California (34.10N, 117.73W), 270 m	PD057
*L*. *pardalinum* (cultivated)	*Pseudolirium*	num	1	USA: Native Botanical Garden of Southern California (34.10N, 117.73W), 270 m	PD056
*L*. *davidii* (cultivated)	*Sinomartagon*	dav	1	China: Kunming Botanical Garden (25.15N, 102.74E), 1900 m	X. Gong PD012
*L*. *davidii* var. *willmottiae*	*Sinomartagon*	wil	1	China: Sichuan (32.07N, 104.30E)	X. Gong PD001
*L*. *duchartrei*	*Sinomartagon*	duc	2	China: Zhongdian, Yunnan (27.88N, 99.66E), 2800 m	X. Gong PD014, PD015
*L*. *leichtlinii* (cultivated)	*Sinomartagon*	lei	1	France: Alpine Botanical Garden du Lautaret (45.03N, 6.40E), 2100 m	L. Malikova PD035
*L*. *taliense*	*Sinomartagon*	tal	2	China: Zhongdian, Yunnan (28.08N, 99.46E), 3300 m	X. Gong PD003, PD004
*L*. *nepalense*	*Sinomartagon*	nep	1	China: Yunnan (25.04N, 102.58)	X. Gong PD009

### Primer design and selection

In total, 29 loci were investigated in our study. Of these loci, 20 were randomly selected from the expressed sequence tags (EST) of *L*. *formosanum* [48] and the NCBI EST database of *L*. *longiflorum* (http://www.ncbi.nlm.nih.gov/nucest). Primers for each selected locus were designed using the software Primer 3.0 [49]. Gene codes, primer sequences, and the putative functions for the 20 EST loci are available in Table 2. In addition, the internal transcribed spacer of the nuclear ribosomal DNA (nrITS) [50], as well as the additional eight chloroplast DNA (cpDNA) loci were employed (Table 2).

**Table 2:** Primers applied in this study.

Gene codes	Amplicon length (bp)	Forward primer (5'-3')	Reverse primer (5'-3')	Putative functions (for ESTs)	Notes
**ESTs**					
Lf108	361–363	AGGATTTCTCGAGGACCTAC	AATCCCAATTTGTATTGCAC	pectinesterase/pectinesterase inhibitor	GW590174.1
Lf207	343–403	ACCCTTTTGTTCCACACGAG	AGATTCCCACTGTCCCTGTCT	cytochrome P450-like TBP protein	GW591169.1
Lf210	302–324	GGGCATTTCATTTTCCCTTT	GAGTACCCACAGGGGTCAAA	S-adenosyl methionine decarboxylase	GW591158.1
Lf212	279–286	GCTCCCCATCACAGTTTCTC	CCACAGGGGTCAAAGTCAAA	S-adenosyl methionine decarboxylase	GW591146.1
Lf218	266	CTGCTCCTGAAGGAATCCAA	GGCTTCGAGACCAACATCAC	LLP-B3 protein	GW591136.1
Lf219	256–299	CTAGGCATCACACCCAAATG	TCCTGATGAAGTCGCTGATG	isopentenyl diphosphate isomerase II (IPI2)	GW591124.1
Lf224	275–299	CTCCTCAATCGGCACATTCT	CAGGGGACTCTCTCTCAGGA	sugar carrier protein	GW591114.1
Lf229	303	CTGCTCCTGAAGGAATCCAA	CGTCGAGGGTCGTGTCTACT	LLP-B3 protein	GW591080.1
Lf230	326	CCATGAGTGGAGTGACATGC	CACACGCTGGACTTCACATT	beta-tubulin	GW591069.1
LL02	526–545	CTCGAGAGAGAGGCCAGATG	CCTCTCTTCCATGCCCATTA	pentatricopeptide repeat-containing protein	DN985104.1
LL17	527	TCTGGAAGTTCTGCCCCTTA	CATCAAACTCCCTCCTCGAC	leucine-rich repeat receptor protein kinase MSP1-like	DN985157.1
LL19	353–496	CAAACCCTTAAACCGCATTG	CAAACCAGTCATTCCCCTGT	fatty acid desaturase	DN985108.1
LL21	539–545	GAGACCCGGCACTGTAAGAC	TGCTGCCAACAAGATAGCAC	hypothetical protein	DN985137.1
LL22	531–536	ATACCAGCAAACTGGGATGC	TCCTTGAGCACAATGAAATCTG	U-box domain-containing protein	DN985128.1
LL25	532–533	TCCGTCTTCCACTTCAGGAT	CACCAACAGCAACAGTCTGG	elongation factor 1-alpha	DR992640.1
LL39	508–539	GACCACCAAAATGTGCAAAA	AGATCAAGAGTCGCCGAAAC	pollen preferential protein	DN985087.1
LL50	493	AGCCAGCAAAGGAATTCTGA	AATTCTCCTCCCCGACATTT	subtilisin-like protease	DN985130.1
LL89	490–491	TTGTTCCACACGAGATTTCTGTT	GACAAGGGGAATCCGACTGT	cytochrome P450-like TBP protein	GR882236.1
LL106	219–220	CAGACTACAATTGAGATYGACTCC	ATCAGGGTTGATGCTCTTGC	heat shock protein 70	BP177599.1
LL107	320	AAGGAGCTTGGGACTGTRATGC	CCTCACTTGGCCATCATGAC	calmodulin	FP052101.1
**cpDNA**					
*rbc*L	500	CGCGGTGGACTTGATTTTACC	GGCATATGCCAAACATGAATACC		(Arzate-Fernández AM et al., 2005)
*psb*C-*trn*S	1467–1470	GGTCGTGACCAAGAAACCAC	GGTTCGAATCCCTCTCTCTC		(Arzate-Fernández AM et al., 2005)
*trn*T-*trn*L	785–818	CAAATGCGATGCTCTAACCT	AGTCCGTAGCGTCTACCGAT		(Nishikawa et al., 2002)
*trn*L-*trn*F	250–251	TCCGTCGACTTTATAAGTCGTG	TGCCAGGAACCAGATTTGAACT		(Nishikawa et al., 2002)
*atp*B-*rbc*L	637–643	GAAGTAGTAGGATTGGTTCTCA	CCAACACTTGCTTTAGTTTC		(Nishikawa et al., 2002)
*pet*A	605–606	CCCATTTTTGCACAGCAGGGTTATG	CCCTCKGAAACAAGAAGTT		(Grivet et al., 2001)
*ycf*4	391–392	GGCCYCGGATTTCCATATAAAG	TGGCGATCAGAACAYATATGGATAG		(Grivet et al., 2001)
*psb*B	628–629	GGATTRCGTATGGGMAATATTGAAAC	CCAAAAGTRAACCAACCCCTTGGAC		(Graham and Olmstead, 2000)
***nrITS***	694–704	GGAAGTAAAAGTCGTAACAAGG	TCCTCCGCTTATTGATATGC		(Peterson et al., 2004)

### PCR, cloning, and sequencing

PCR amplification was conducted with a reaction volume of 50 μL, containing 25 μL of 2×*Taq* polymerase master mix (Ampliqon, Denmark), 5 μL of template DNA (2 ng/μL), 5 μL of each primer (2 pM), and distilled water. The PCR reactions were performed using the MyCycler thermal cycler (Bio-Rad, USA) with 35 cycles. For each cycle, we set an initial denaturation at 94°C for 50 s, annealing at 48–53°C (optimized for each locus, Table 2) for 50 s, and extension at 72°C for 80 s. A final extension at 72°C for 10 min was applied. The PCR products were run on agarose gels, and the targeted DNA fragments were sliced and purified. The purified PCR products were ligated to a pGEM-T Easy vector at 4°C overnight and transformed into *E*. *coli DH5a* cells. Positive clones were validated with blue-white screening followed by colony PCR. To ensure that both diploid alleles were sequenced, we randomly selected five to seven clones from each individual, and discarded the low-frequency clones due to possible PCR errors. Sanger sequencing was conducted in both directions with the universal T7P and SP6 primers using the 96-capillary 3730xl DNA Analyzer (Genomics Biotech Co., Ltd.).

### Sequence alignments, indel identification, and tree reconstructions

The sequences of the EST, nrITS, and cpDNA loci of the 20 *Lilium* species were validated using BLAST on NCBI. All sequences were deposited in the NCBI GenBank, with accession numbers of KX863745-KX865072. In addition, the full-length chloroplast genome and nrITS sequences of *Cardiocrinum cordatum* (KX575837.1 and KP712019.1, respectively) and *Fritillaria taipaiensis* (KC543997.1 and KT861551.1, respectively) were downloaded from NCBI as outgroups for the *Lilium* species. Sequences of each locus were then aligned using CLC Free Workbench (http://www.clcbio.com/) with the default settings, and gap sites were manually checked. Indel events for each locus were identified and coded with SeqState [51] to be incorporated in the phylogeny reconstruction. Genetic distances of each alignment were estimated using the two-parameter model implemented in MEGA 6 [52–53].

For the cpDNA markers, the alignments of all the genes representing 5,432 bp were concatenated, the indels were coded according to Simmons and Ochoterena’s simple coding method [54] in SeqState, and a Bayesian inference tree was generated with MrBayes v. 3.2.6 [55]. The best substitution models for the cpDNA were evaluated by MEGA6, and the substitution model used in MrBayes was set accordingly (S1 Table). We performed > 100,000 steps of a Markov chain Monte Carlo (MCMC) for each gene to ensure the average standard deviation of the split frequencies was lower than 0.01, with a sample frequency of 100, print frequency of 100, and diagnosis frequency of 1,000. After summarizing the parameter values, the potential scale reduction factor was confirmed to be approximately 1.0 for all parameters. Finally, the consensus tree was summarized using the default settings. For nrITS and EST markers, tree reconstructions were also conducted with MrBayes for individual loci with the same settings as the cpDNA.

For integrating the information of cpDNA and nrITS genes to reconstruct the phylogeny, the software BEAST version 1.7.5 [56] was used. An uncorrelated relaxed clock model was set for both cpDNA and nrITS loci. The priors of the substitution rate were set as uniform distributions at initial values of 0.000933 and 0.00968, respectively. The prior of the divergence time of *Lilium* samples was set as a normal distribution with a mean of 13.6 million years and a standard deviation of 1.5 million years based on the estimation of Gao *et al*. [57]. The length of the MCMC run was set to 10 million, and the parameters were saved every 1,000 steps. The results of the trees for 10 independent runs with different seeds were combined with the program logcombiner [56], with 50% of the trees discarded as burn-in, and subsequently processed by the program treeannotator [56] to generate a consensus tree (hypothetical species tree). The hypothetical species tree was visualized using FigTree v. 1.4.2. (http://tree.bio.ed.ac.uk/software/figtree/), converted to the Newick format, and then annotated in MEGA6.

### Intraspecific genetic variations, recombination rates (Rm)

Genetic diversities among the studied taxa were estimated using DnaSP version 5.0 [58]. The nucleotide diversities (π) and the minimum recombination events were calculated. The potential gene flow among the species was inferred from the recombination events and the shared genetic variations among the species [59].

### IMa analyses

To sophisticatedly estimate gene flow between species clusters, the Isolation with Migration model that was implemented in program IMa2 [60] was applied, and six model parameters were calculated using coalescence simulations and Bayesian computational procedures: the divergence time (*t*), the bidirectional migration rates (*m*_1_ and *m*_2_), and the effective population sizes of the ancestral (*θ*_A_) and two current populations (*θ*_1_ and *θ*_2_). Taxa with a possible hybrid origin, *L*. *davidii var*. *willmotiae*, *L*. *bulbiferum*, and *L*. ‘*Casa Blanca*’, were excluded. Using the coalescence time of 13.6 million years ago for the *Lilium* crown group [57], we estimated the substitution rates for the 20 EST loci by dividing the root height of the Bayesian trees by the coalescence time (S2 Table). The infinite site (IS) models were applied to all the loci. Because the IMa2 program does not accept genes containing recombinant fragments, the IMGC program was used to extract the largest non-recombinant DNA fragments from the aligned sequences [61]. After processing our data using the IMGC program, the DNA fragments that were shorter than 100 bp were excluded due to a lack of sufficient genetic information. To ensure that there were enough heating steps for obtaining a reliable result, 20 Markov chain Monte Carlo chains were used with the following heating parameters: ha of 0.96 and hb of 0.9. IMa2 runs were performed and saved using at least 3 million burn-in steps followed by at least 5 million steps (50,000 genealogies). All the effective parameter sample sizes were greater than 100. Three independent runs with different random seeds were performed to check for consistency across the results. The final results were calculated by averaging the values estimated in each run. The migration rate per generation (*M*) was calculated by multiplying the *m* value by the geometric mean of the substitution rates (*μ*).

## Results

### Phylogeny based on eight cpDNA genes

**Fig 1:**
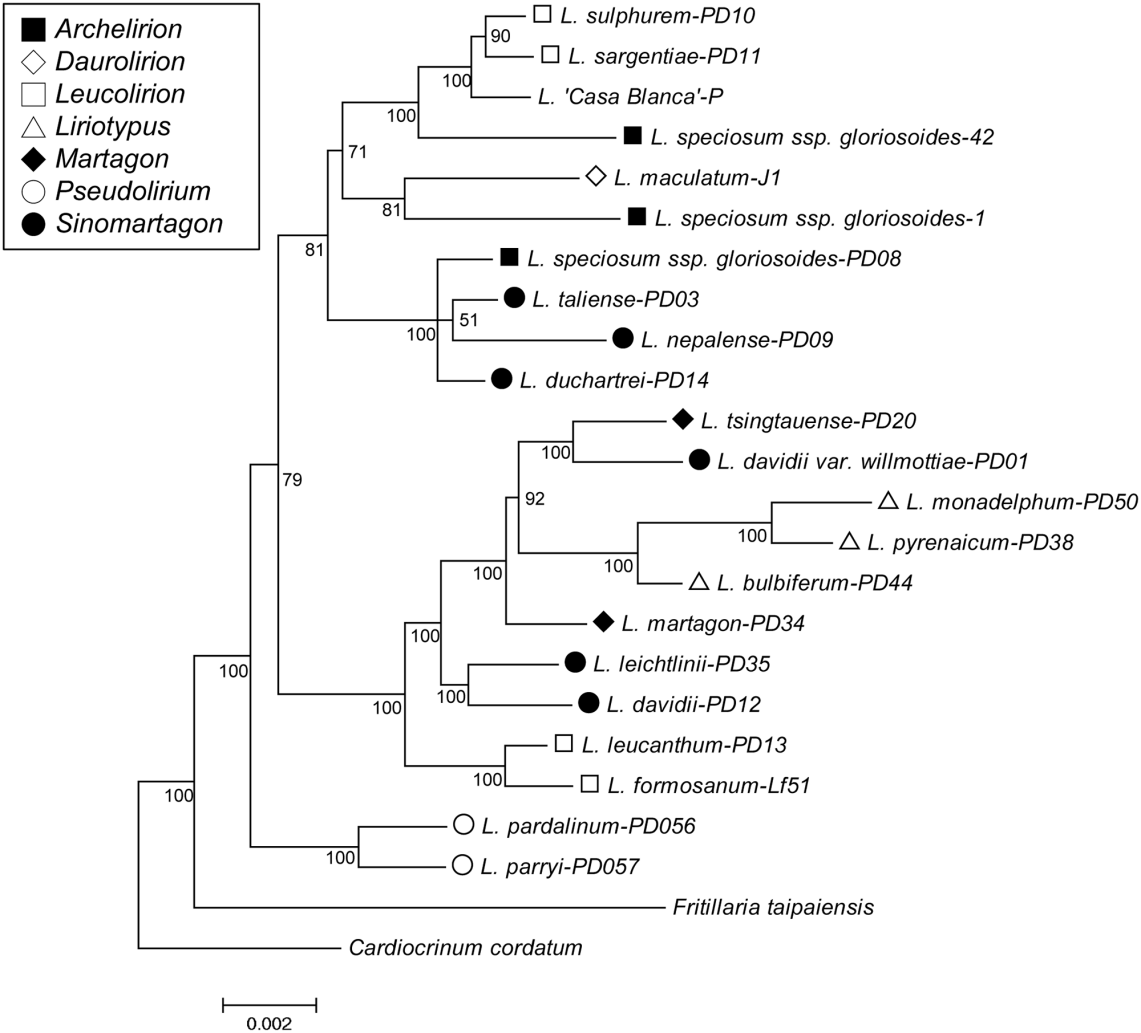
Bayesian inference phylogram reconstructed using the concatenation of eight cpDNA genes with MrBayes. The tree was rooted at *Cardiocrinum cordatum*. Sections are coded as follows: section *Archelirion*, solid square, section *Daurolirion*, blank diamond, section *Leucolirion*, blank square, section *Liriotypus*, blank triangle, section *Pseudolirium*, blank circle, section *Sinomartagon*, solid circle, and section *Martagon*, solid diamond. Posterior probabilities are shown below the branches.

The Bayesian inference phylogram based on the concatenation of eight cpDNA loci showed that the *Pseudolirium* section was monophyletic and at the basal position, while 18 other species from 5 sections were clustered into two main groups (Fig 1). The *Leucolirion*, *Martagon*, and *Sinomartagon* sections were paraphyletic or polyphyletic, whereas the *Liriotypus* section was monophyletic. Unexpectedly, three *Lilium speciosum* var. *gloriosoides* samples from different populations (two samples from Taiwan and one from Yunnan in China, see Table 1) were not clustered together. Within the *Leucolirium* section, *L*. *formosanum* was sister to *L*. *leucanthum*, *L*. *sulphureum* was sister to *L*. *sargentiae*, but the four taxa were not clustered together. Within the *Sinomartagon* section, *L*. *leichtlinii* was clustered with *L*. *davidii* var. *davidii*, but *L*. *taliense*, *L*. *duchartrei*, and *L*. *nepalense* were clustered with *L*. *speciosum* var. *gloriosoides* from Yunnan (PD08) of the *Archelirion* section. In addition, *L*. *davidii* var. *willmottiae* of the *Sinomartagon* section was sister to *L*. *tsingtauense* in the *Martagon* section, suggesting a hybrid origin.

### Phylogeny based on nrITS

**Fig 2:**
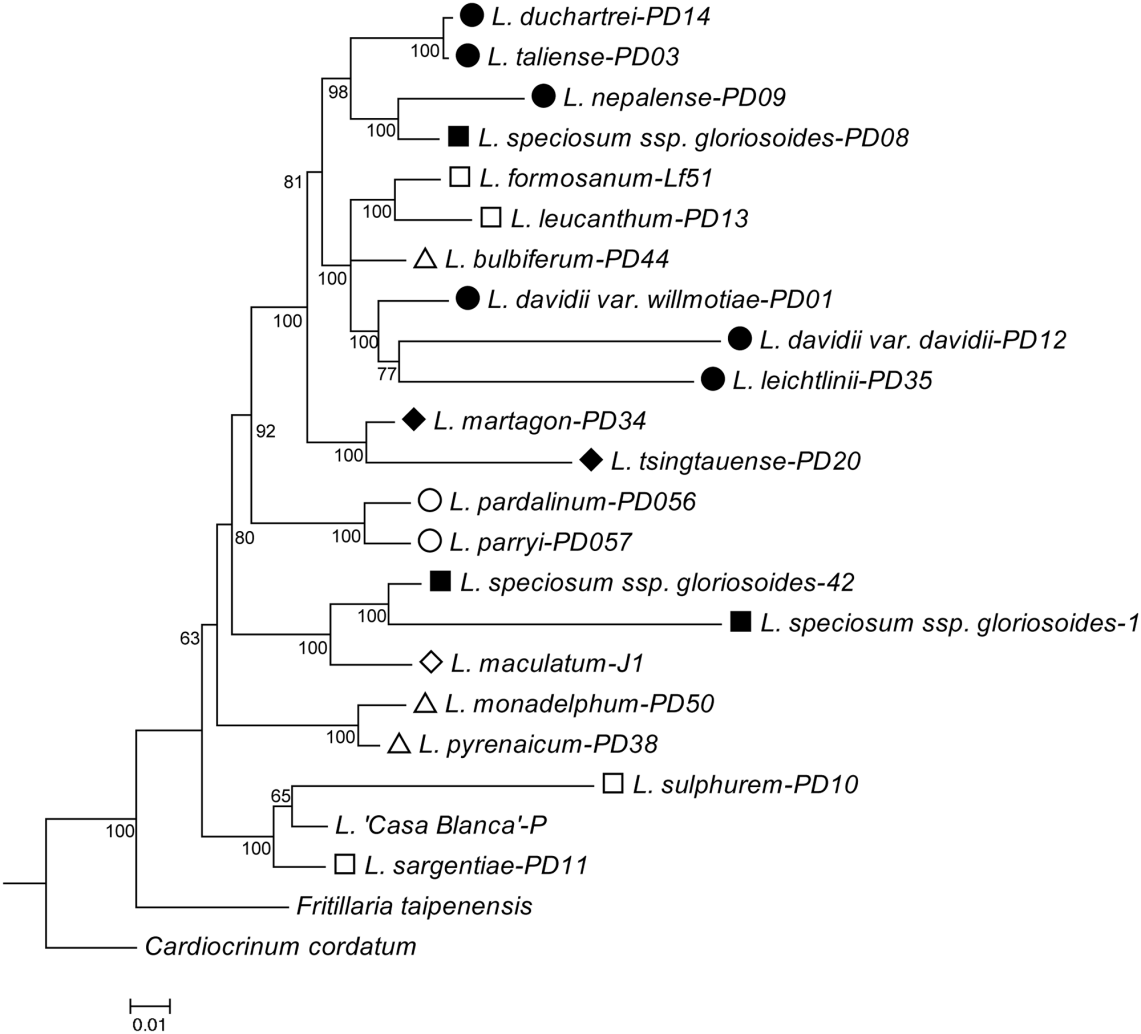
Bayesian inference phylogram reconstructed based on nrITS with MrBayes. The tree was rooted at *Cardiocrinum cordatum*. Posterior probabilities are shown below the branches. The sections are coded the same as they are in Fig 1.

The Bayesian inference tree based on nrITS suggested that 20 *Lilium* species were divided into one main cluster and several smaller clusters (Fig 2). The main cluster consisted of six species of section *Sinomartagon*, *L*. *formosanum* and *L*. *leucanthum* of section *Leucolirion*, *L*. *bulbiferum* of section *Liriotypus*, and *L*. *speciosum* ssp. *gloriosoides* (section *Archelirion*) from Yunnan. For the small clusters, two species in the *Martagon* section were clustered, two species in the *Pseudolirium* section were grouped together, *L*. *speciosum* ssp. *gloriosoides* (section *Archelirion*) from Taiwan was clustered with *L*. *maculatum* in section *Daurolirion*, *L*. *monadelphum* and *L*. *pyrenaicum* in section *Liriotypus* were clustered, while *L*. *sulphureum* and *L*. *sargentiae* in section *Leucolirion* were clustered with the cultivar *L*. *‘Casa Blanca’*. The ITS topology displayed a closer relationship between *L*. *bulbiferum* and section *Sinomartagon*, which has also been revealed in previous studies [38,40,57].

### Phylogeny based on the combined data of nrITS and cpDNA genes: Hypothetical species tree

**Fig 3:**
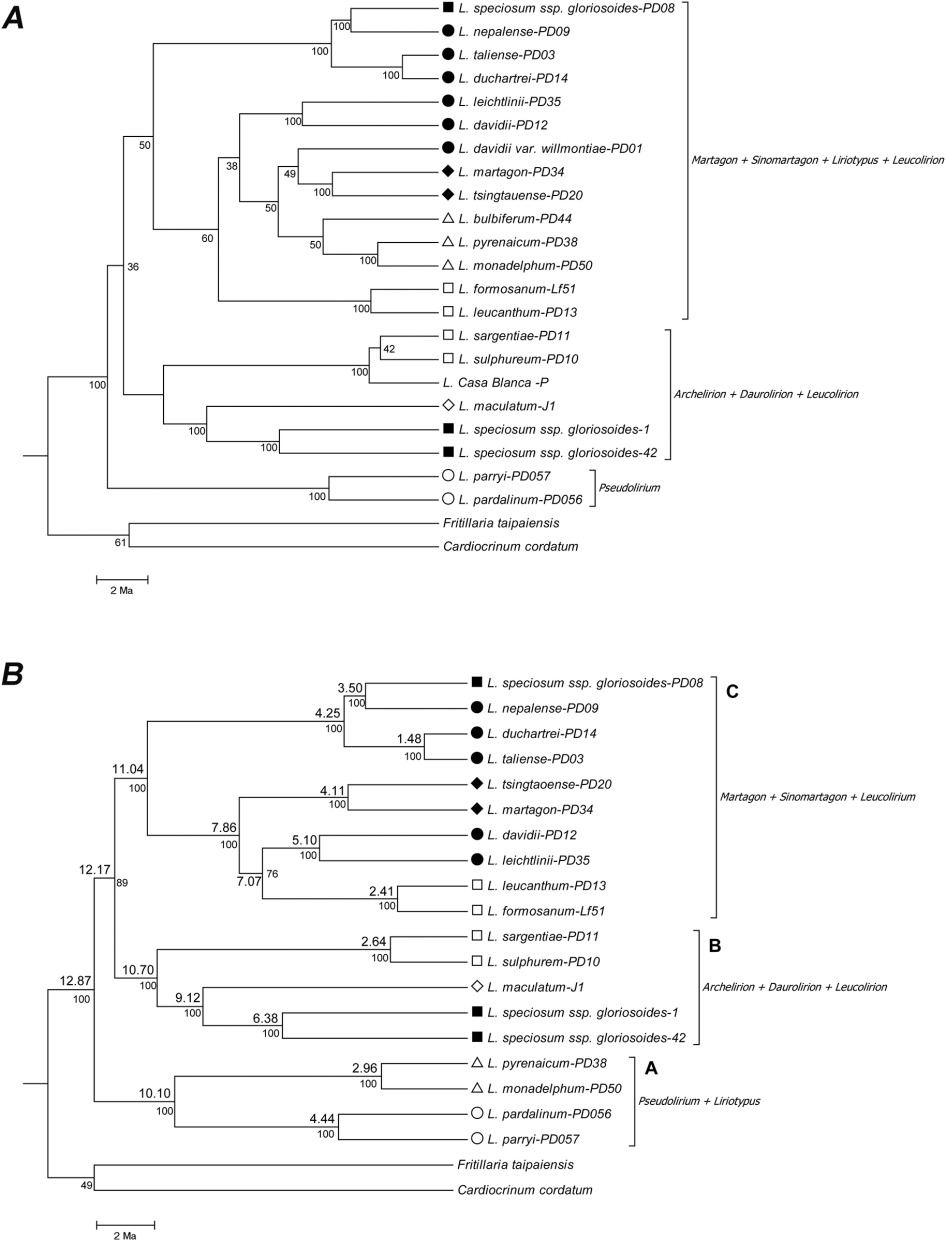
The phylogeny reconstructed based on the combined data of nrITS and cpDNA genes with the BEAST analysis. The tree was rooted at the outgroups of *Fritillaria taipaiensis* and *Cardiocrinum cordatum*. The sections are coded as they are in Fig 1. The scale bar denotes 2 million years ago (Ma). Posterior probabilities are shown below the branches. (A) All sampled taxa were analyzed. (B) *L*. *bulbiferum*, *L*. *davidii* var. *willmotiae*, *L*. ‘*Casa Blanca*’ were removed from the analysis due to their possible hybrid origins. The tree was deemed the hypothetical species tree. Numbers on the branches represent estimated divergence times.

By combining nrITS and cpDNA regions using the BEAST software, the phylogeny showed better resolution on the delimitation of the sections (Fig 3A). This tree uncovered three clusters: Cluster A of *L*. *pardalinum* and *L*. *parryi* (sect. *Pseudolirium*); Cluster B of *L*. *sargentiae*, *L*. *sulphureum* (sect. *Leucolirium*), *L*. *maculatum* (sect. *Daurolirion*), *L*. *speciosum* ssp. *gloriosoides* in Taiwan (sect. *Archelirion*), and *L*. '*Casa Blanca*'; and Cluster C comprising *L*. *martagon*, *L*. *tsingtauense* (sect. *Martagon*), *L*. *davidii*, *L*. *davidii* var. *willmottiae*, *L*. *leichtlinii*, *L*. *taliense*, *L*. *duchartrei*, *L*. *nepalense* (sect. *Sinomartagon*), *L*. *formosanum*, *L*. *leucanthum* (sect. *Leucolirium*), *L*. *bulbiferum*, *L*. *pyrenaicum*, *L*. *monadelphum* (sect. *Liriotypus*), and *L*. *speciosum* ssp. *gloriosoides* in China (sect. *Archelirion*). When *L*. *davidii* var. *willmotiae*, *L*. *bulbiferum*, and *L*. ‘*Casa Blanca*’ with a possible hybrid origin were removed from the analysis, the *Liriotypus* section clustered with the *Pseudolirium* section of Cluster A instead of the *Martagon* and *Sinomartagon* sections of Cluster C, while the other taxa remained in the previous positions, implying an affinity between *L*. *bulbiferum* and the *Sinomartagon* section (Fig 3B). This tree without the hybrid interference better resolved the phylogenetic relationships among the *Lilium* sections. Here, we identified nine sister groups. They were *L*. *formosanum* and *L*. *leucanthum*, *L*. *sargentiae* and *L*. *sulphureum* (sect. *Leucolirion*), *L*. *davidii* var. *davidii* and *L*. *leichtlinii*, *L*. *taliense* and *L*. *duchartrei*, *L*. *nepalense* (sect. *Sinomartagon*) and *L*. *speciosum* var. *gloriosoides* in China (sect. *Archelirion*), *L*. *tsingtauense* and *L*. *martagon* (sect. *Martagon*), *L*. *pyrenaicum* and *L*. *monadelphum* (sect. *Liriotypus*), *L*. *pardalinum* and *L*. *parryi* (sect. *Pseudolirium*), as well as *L*. *speciosum* var. *gloriosoides* in Taiwan and *L*. *maculatum* (sects. *Archelirion* and *Daurolirion*). Hence, the tree was deemed the hypothetical species tree (Fig 3B) in the following discussions. Molecular dating with BEAST revealed that the coalescence time of *Lilium* was approximately 12.87 million years ago (Ma) (95% CI: 9.88–15.81). The coalescence time of Clusters A, B, and C were dated at 10.10 Ma (95% CI: 6.88–13.33), 10.70 Ma (95% CI: 7.87–13.87), and 11.04 Ma (95% CI: 8.08–14.08), respectively, and the coalescence times of the nine sister groups ranged from 1.48 Ma (*L*. *duchartrei* and *L*. *taliense*, 95% CI: 0.41–3.16) to 9.12 Ma (*L*. *maculatum* and *L*. *speciosum* ssp. *gloriosoides* of Taiwan, 95% CI: 7.87–13.87), suggesting long divergences among these *Lilium* species (Fig 3B).

Furthermore, the topology comparisons between the hypothetical species tree (Fig 3B) and the 20 EST trees (S1 Appendix) were conducted focusing on the sister relationships among the groups. Of the gene phylogenies, only the *LL22* tree uncovered all the sister groups (S1 Appendix). Of these sister groups, *L*. *pardalinum* and *L*. *parryi* showed the highest supporting rate (55%), followed by *L*. *formosanum* and *L*. *leucanthum* (40%) as well as *L*. *sargentiae* and *L*. *sulphureum* (40%) (S3 Table). Unexpectedly, the sister relationship between *L*. *speciosum* ssp. *gloriosoides* in Taiwan and *L*. *maculatum* was not supported by any EST tree.

### Intraspecific genetic variations, recombination events, and IMa analyses

The nucleotide diversities of the chloroplast genes among the 20 *Lilium* species were generally lower than those of the nuclear loci. Locus Lf207 had the lowest nucleotide diversities (π = 0.01527 ± 0.00322), while the nucleotide diversities of Lf210, Lf224, LL19, LL25, LL89, LL106, and nrITS were relatively higher (Table 3). Moreover, fewer recombination events were detected in the chloroplast loci (Table 3). It is likely attributable to maternal inheritance of the chloroplast DNA, which reserved the primordial genetic variation of the ancestral genotypes. In contrast, high recombination rates were detected in most of the EST loci, i.e., *Lf108*, *Lf207*, *Lf210*, *Lf212*, *Lf224*, *Lf229*, *LL02*, *LL17*, *LL19*, *LL22*, *LL25*, *LL39*, *LL50*, *LL89*, *LL106*, and *LL107*.

**Table 3:** Recombination rates and nucleotide diversities of the 20 *Lilium* species.

Gene codes	Minimum numbers of recombination events (Rm)	Nucleotide diversity (π)
Lf108	16	0.05714±0.00213
Lf207	12	0.01527±0.00322
Lf210	23	0.05868±0.00626
Lf212	15	0.05205±0.00456
Lf218	9	0.03955±0.00265
Lf219	7	0.03979±0.00171
Lf224	28	0.14724±0.01027
Lf229	11	0.03906±0.00233
Lf230	6	0.02865±0.00279
LL02	14	0.04163±0.00193
LL17	15	0.03498±0.00151
LL19	20	0.07564±0.00829
LL21	3	0.01854±0.00095
LL22	13	0.03825±0.0014
LL25	23	0.07758±0.00654
LL39	17	0.05378±0.00144
LL50	19	0.05061±0.00125
LL89	28	0.0212±0.00486
LL106	30	0.17965±0.00755
LL107	13	0.03966±0.00674
ITS	52	0.08574±0.0089
*rbc*L	1	0.00623±0.00058
*psb*C-*trn*S	3	0.00824±0.00055
*trn*T-*trn*L	3	0.01754±0.00115
*trn*L-*trn*F	0	0.00691±0.00137
*atp*B-*rbc*L	0	0.00671±0.00096
*pet*A	0	0.00678±0.00078
*ycf*4	0	0.00865±0.00318
*psb*B	0	0.00586±0.00068

**Fig 4:**
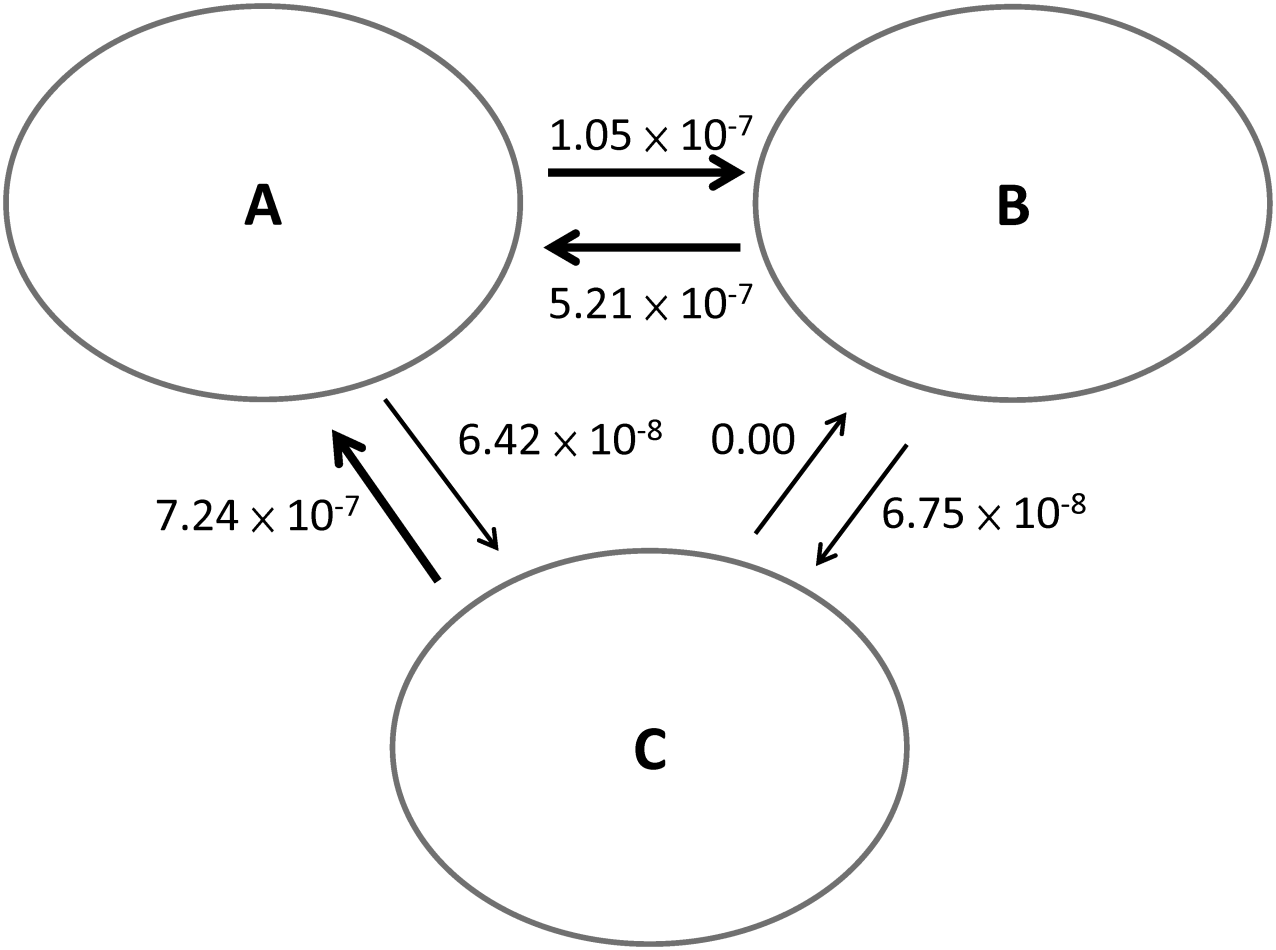
Gene flow among the three clusters suggested by the hypothetical species tree. Numbers denote the extent of the gene flow estimated by the IMa2 analysis. The thick arrow indicates significant gene flow as suggested by the likelihood ratio tests.

We used IMa2 to evaluate the levels of gene flow among the three *Lilium* clusters (Fig 4). The taxa that had a possible hybrid origin were excluded from this analysis. The level of gene flow was estimated by the migration rates per generation (*M*). In the pairwise comparisons between Clusters A, B, and C, the highest level of gene flow occurred from Clusters C to A (*M* = 7.43 × 10^−7^), followed by the gene flow from Clusters B to A (*M* = 5.21 × 10^−7^) and the gene flow from Clusters A to B (*M* = 1.03 × 10^−7^). All the above directions showed significant gene flow, as was suggested by the likelihood ratio test (p < 0.05) (S4 Table). These results suggested that hybridization across sections may have occurred frequently in *Lilium*.

### Indel events

Our results revealed that indel distributions varied among the *Lilium* taxa in the nuclear loci. The indel events in cpDNA and nrITS were identified and were included in the phylogeny reconstruction, and some informative indels in the EST loci were shown in the S5 Table. For example, a 12-bp insertion at site 20 on *LL39* was found in *L*. *tsingtauense* and *L*. *martagon* (sect. *Martagon*), and *L*. *taliense* and *L*. *duchartrei* (sect. *Sinomartagon*) shared a 1-bp deletion at site 458 on *LL22*. Some indels were shared by multiple sections. For instance, a 13-bp deletion at site 519 on *LL39* occurred in the *Pseudolirium* (*L*. *pardalinum* and *L*. *parryi*) and *Leucolirion* sections (*L*. *sulphureum* and *L*. *sargentiae*), and a 3-bp deletion at site 264 of *LL39* was found in *L*. *speciosum* var. *gloriosoides* (sect. *Archelirion*) and *L*. *nepalense* (sect. *Sinomartagon*).

## Discussion

This study unraveled the factors that contribute to the phylogenetic conflicts by exploring multilocus phylogenies of *Lilium*. Although phylogenetic conflict can be rampant across loci, only a few studies have addressed the causal factors comprehensively [3–4]. For *Lilium*, several phylogenetic studies reflected its phylogenetic conflict but failed in illustrating the mechanism or factors resulting in the conflict [41–42]. Here, we evaluated the influences of the analytical and biological factors that led to the phylogenetic conflict in *Lilium*.

### Analytical and biological factors causing phylogenetic conflicts

#### Multilocus approach

While the debate on the advantages and disadvantages of combining data in reconstructing multilocus phylogeny continues, many recent studies suggested that combining all the available data is feasible and reliable and that elucidating incongruence provides hints into the evolutionary history [21,23,25]. Unfortunately, most of the phylogenetic studies that combined sequences across loci provided very few explanations regarding the reliability of the combined trees (e.g., [62–63]). In our study, with a wide locus sampling, predominant phylogenetic incongruences across different loci were revealed (e.g., S3 Table and S1 Appendix). By combining the cpDNA and nrITS loci, species from the same sections were mostly clustered and the topology of the 17 *Lilium* species substantially agreed with the taxonomic sections by Comber [30] (Fig 3B). The hypothetical species tree (Fig 3B) uncovered three clusters: Cluster A (sect. *Pseudolirium and Liriotypus*), Cluster B (*Leucolirion*, *Daurolirion*, and *Archelirion*), and Cluster C (*Leucolirion*, *Martagon* and *Sinomartagon*). The multilocus analyses not only gave an insight into the *Lilium* phylogeny but also provided opportunities to uncover the taxa that had a hybrid origin as shown earlier and revealed the interspecific gene flow, which is addressed in the following paragraphs.

#### Interspecific gene flow

Intraspecific gene flow could provide concordance in the species genome. It would homogenize the genomes and thereby block the genome divergence of the isolated populations [64]. However, when gene flow among taxa occurs, phylogenetic incongruence ineluctably arises [14–15]. Interspecific gene flow is not rare in plants [65,66]. Examples include *Howea belmoreana* and *H*. *forsteriana* [17], and *Arabidopsis halleri* and *A*. *lyrata* [18], all revealing uninterrupted gene flow after speciation [18]. Interspecific gene flow causes extreme difficulty with regard to the phylogenetic reconstruction.

The divergence of the crown group of *Lilium* can be dated back to 13.6 million years ago [57]. All the examined taxa in our study diverged a very long time, even the closest sister pair, *L*. *taliense* and *L*. *duchartrei*, for whom the divergence was more than 1.4 million years. Of the three clusters identified in the hypothetical species tree (Fig 3B), long divergences between the clusters (more than 10 million years) tended to reject incomplete lineage sorting, which blurs the species delimitation in *Lilium*. Given the rampant phylogenetic incongruence across the loci, interspecific gene flow may have been largely involved in the evolution of the *Lilium* species. Here, three inspections were proposed to elucidate the possibility and strength of interspecific gene flow.

First, as demonstrated earlier, *L*. *bulbiferum* and *L*. *davidii* var. *willmottiae* were recognized as hybrids based on their inconsistent placements on the cpDNA and nrITS trees (Figs 1 and 2). Second, in the DnaSP analysis, 17 out of 20 (85%) EST loci appeared to have high number of recombination events (Table 3). Third, the result of the IMa2 analyses suggested that historical gene flow likely occurred between the three clusters of the hypothetical species tree, especially the genetic exchanges with Cluster A (Fig 4). It is noticeable that all the samples in Cluster A were cultivars, implying the possibility of artificial hybridization, whether intentional or not. It has been shown that the artificial crosses between the different *Lilium* sections were common and not difficult [67]. As the strongest gene flow occurred from Cluster C, which predominately originated from China, to Cluster A of America or Europe, the gene flow across continents also implied that artificial hybridization may have blurred the species/section boundaries of the *Lilium* species.

Overall, even though our inspections have limited power in surveying the quantity and direction of gene flow due to the small sample size of each taxon, our results suggested that extensive gene flow among taxa had occurred in *Lilium*. The plentiful interspecific gene flow apparently contributed to the difficulties in section delimitation. It was likely that interspecific gene flow arose from artificial hybridizations, and thereby caution ought to be exercised in using cultivated samples for phylogeny reconstruction.

### Phylogenetic implications

Our results reflected adequate resolution on the phylogenies, despite a small sample size, reflecting the power of incorporating multiple loci in the phylogenetic reconstruction. Some taxa that were assigned to the same section appeared to be sister groups in the phylogenies of cpDNA and nrITS, thanks to the low genetic recombination and lower substitution rate of the maternally inherited cpDNA (Table 3 and S2 Table; [68]) and/or the concerted evolution of nrITS [69–71].

Our phylogenies affirmed the previous studies that identified the *Martagon* section as a monophyletic group [37–38,43–44] with close relationships to the *Leucolirion* and *Sinomartagon* sections [36] (Fig 3B). Moreover, the *Liriotypus* section was determined to be polyphyletic in previous studies that include *L*. *bulbiferum* [38,40,43], whereas it was determined to be monophyletic in the studies that excluded *L*. *bulbiferum* from the analysis [37,44]. Our study revealed similar results, with the cpDNA phylogeny uncovering monophyly of the *Liriotypus* section, whereas the nrITS phylogeny showed that *L*. *bulbiferum* clustered with the *Sinomartagon* section. Apparently, the hybrid origin of *L*. *bulbiferum* caused noise in the phylogenetic inference. Likewise, the BEAST analysis, which was based on the combined data of cpDNA and nrITS, further supported the monophyly of the *Liriotypus* section and its close affinity to the *Sinomartagon* section. Interestingly, when *L*. *bulbiferum* was removed from the phylogenetic analysis, the *Liriotypus* section became the neighbor of the *Pseudolirium* section (Fig 3B). Furthermore, phylogenies of the EST loci revealed that *L*. *bulbiferum* was clustered with *L*. *davidii*, *L*. *leichtlinii* (sect. *Sinomartagon*), *L*. *monadelphum*, or *L*. *pyrenaicum* (sect. *Liriotypus*) (S1 Appendix). These results suggested that *L*. *bulbiferum* did show affinity to the *Sinomartagon* section, as suggested by other studies based on nrITS [38;40;57] and a maternal background from the *Liriotypus* section based on the chloroplast DNA. Altogether, we suggested that *L*. *bulbiferum* is likely a hybrid between the *Sinomartagon* and *Liriotypus* section, with the latter as the maternal parent. Furthermore, the *Pseudolirium* section appeared to be basal in *Lilium*, both in the phylogenies of cpDNA and the combined data, a finding consistent with the *matK* gene phylogeny [57].

Our study also revealed that the *Leucolirion* and *Sinomartagon* sections were polyphyletic, which largely corroborated earlier phylogenies [36–38,43–44, 57]. In the *Leucolirion* section, 40% of the EST loci supported that *L*. *formosanum* and *L*. *leucanthum* were sisters, and 40% of nuclear loci showed that *L*. *sargentiae* was related to *L*. *sulphureum*, while no data collected here indicated the clustering of these four species (S3 Table, S1 Appendix). The close relationship between *L*. *sargentiae* and *L*. *sulphureum* also corresponded to the geographic regions where they grow. In contrast, no overlap in the distribution of *L*. *formosanum* and *L*. *leucanthum* has been reported. Although the allopatric distribution of this sister group might imply a longer divergence between *L*. *formosanum* and *L*. *leucanthum*, the close phylogenetic relationship indicated their affinity. However, the possibility of a sampling bias that contributes to this allopatric match relationship cannot be ruled out. The cpDNA, nrITS, and the combined data all suggested that *L*. *‘Casa Blanca’* was clustered with *L*. *sargentiae* and *L*. *sulphureum* (Figs 1–3). This may infer that part of the parental species of *L*. *Casa Blanca* was from the *Leucolirion* section. The largest section, *Sinomartagon*, contains 22 taxa, which are morphologically distinguishable from each other [30,72]. Nishikawa *et al*. [38] divided this section into five groups according to the phylogeny based on the nrITS data. Our results on the *Sinomartagon* section generally agreed with Nishikawa *et al*.’s work [38].

Even though our sampling on the *Archelirion* section was restricted to two populations of *L*. *speciosum* ssp. *gloriosoides*, the hypothetical species tree and most of the EST trees revealed genetic dissimilarities between the populations (Fig 3B and S1 Appendix). The individuals in Taiwan were closely related to the *Daurolirion* section, while the individual isolated from China was related to *L*. *nepalense* of the *Sinomartagon* section. Accordingly, we suggest assigning *L*. *speciosum* ssp. *gloriosoides* of China to the *Sinomartagon* section instead of the *Archelirion* section.

## Conclusions

In summary, multilocus analyses enabled us to uncover interspecific gene flow, identify the taxa with hybrid origins, and comprehensively reconstruct the evolutionary history of *Lilium*. The hypothetical species tree better resolved the section classification of the *Lilium* species. Our study suggested that future studies exploring both analytical and biological factors that cause phylogenetic conflicts would provide a better understanding of the evolutionary relationship among plant species.

## Supporting information

S1 Table The substitution models for all the loci used in this study. (DOCX)

S2 Table The substitution rates of the 20 EST loci used in the IMa2 analysis. (DOCX)

S3 Table Topology comparisons between the hypothetical species tree and the 20 EST trees. (DOCX)

S4 Table Summary statistics of the migration rates estimated by IMa2. (DOCX)

S5 Table Summary of the indel-sharing events. (DOCX)

S1 Appendix Bayesian inference phylogenies of the 20 EST loci. (PDF)
