# Supplementary material for: Frequent gene flow blurred taxonomic boundaries of sections in Lilium L. (Liliaceae)
Source: PLoS One. 2017 Aug 25;12(8):e0183209. doi: 10.1371/journal.pone.0183209 (PMC5571923; doi:10.1371/journal.pone.0183209)
Supplement: S1 Table — (DOCX) [file pone.0183209.s001.docx]

**S1 Table.** The substitution models for all loci used in this study.

| **Gene** | **Substitution Models** |
| --- | --- |
| Lf108 | K2P+G |
| Lf207 | K2P+G |
| Lf210 | K2P+G |
| Lf212 | K2P+G |
| Lf218 | K2P+G |
| Lf219 | K2P+G |
| Lf224 | K2P+G |
| Lf229 | K2P+G |
| Lf230 | K2P+G |
| LL02 | HKY+G |
| LL17 | K2P+G |
| LL19 | K2P+G |
| LL21 | K2P |
| LL22 | K2P+G |
| LL25 | K2P+G |
| LL39 | K2P+G |
| LL50 | K2P+G |
| LL89 | K2P+G |
| LL106 | K2P+G |
| LL107 | K2P+G |
| cpDNA | HKY+G |
| nrITS | K2P+G |
