# Supplementary material for: Frequent gene flow blurred taxonomic boundaries of sections in Lilium L. (Liliaceae)
Source: PLoS One. 2017 Aug 25;12(8):e0183209. doi: 10.1371/journal.pone.0183209 (PMC5571923; doi:10.1371/journal.pone.0183209)
Supplement: S2 Table — (DOCX) [file pone.0183209.s002.docx]

**S2 Table.** The substitution rates of 20 EST loci used in IMa2 analysis.

| **Gene** | **Number of mutations per gene per generation** |
| --- | --- |
| Lf108 | 0.00000114 |
| Lf207 | 0.00000079 |
| Lf210 | 0.00000244 |
| Lf212 | 0.00000152 |
| Lf218 | 0.00000066 |
| Lf219 | 0.00000053 |
| Lf224 | 0.00000349 |
| Lf229 | 0.00000071 |
| Lf230 | 0.00000060 |
| LL02 | 0.00000126 |
| LL17 | 0.00000096 |
| LL19 | 0.00000364 |
| LL21 | 0.00000048 |
| LL22 | 0.00000136 |
| LL25 | 0.00000400 |
| LL39 | 0.00000192 |
| LL50 | 0.00000130 |
| LL89 | 0.00000254 |
| LL106 | 0.00000429 |
| LL107 | 0.00000166 |
