# Supplementary material for: Frequent gene flow blurred taxonomic boundaries of sections in Lilium L. (Liliaceae)
Source: PLoS One. 2017 Aug 25;12(8):e0183209. doi: 10.1371/journal.pone.0183209 (PMC5571923; doi:10.1371/journal.pone.0183209)
Supplement: S3 Table — (DOCX) [file pone.0183209.s003.docx]

**S3 Table** Topology comparisons between the hypothetical species tree and the 20 EST trees.

| **Species clusters** | **Supporting rate** | **Sections** |
| --- | --- | --- |
| Sister groups^1^ |  |  |
| (tsi, mar) | 20% | *Martagon* |
| (num, ryi) | 55% | *Pseudolirium* |
| (for, leu) | 40% | *Leucolirion* |
| (sar, sul) | 40% | *Leucolirion* |
| (pyr, mon) | 15% | *Liriotypus* |
| (tal, duc) | 15% | *Sinomartagon* |
| (nep, gl8) | 30% | *Sinomartagon* |
| (dav, lei) | 10% | *Sinomartagon* |
| (spe, mac) | 0% | *Archelirion* & *Daurolirion* |

^1^based on the hypothetical species tree
