# Supplementary material for: Frequent gene flow blurred taxonomic boundaries of sections in Lilium L. (Liliaceae)
Source: PLoS One. 2017 Aug 25;12(8):e0183209. doi: 10.1371/journal.pone.0183209 (PMC5571923; doi:10.1371/journal.pone.0183209)
Supplement: S4 Table — (DOCX) [file pone.0183209.s004.docx]

**S4 Table. Summary statistics of migration rates estimated by IMa2**.

.

| A vs B | m(B to A) | | | | m(A to B) | | | |
| --- | --- | --- | --- | --- | --- | --- | --- | --- |
|  | Lower 95% | Peak | Higher 95% | LLRtest | Lower 95% | Peak | Higher 95% | LLRtest |
| run1 | 0.000 | 0.075 | 0.325 | 2.914* | 0.095 | 0.371 | 0.625 | 13.252*** |
| run2 | 0.000 | 0.074 | 0.325 | 2.848* | 0.095 | 0.371 | 0.625 | 9.269** |
| run3 | 0.000 | 0.076 | 0.315 | 2.351 | 0.105 | 0.371 | 0.635 | 8.187** |
| A vs C | m(C to A) | | | | m(A to C) | | | |
|  | Lower 95% | Peak | Higher 95% | LLRtest | Lower 95% | Peak | Higher 95% | LLRtest |
| run1 | 0.185 | 0.514 | 1.015 | 12.479*** | 0.000 | 0.044 | 0.215 | 2.277 |
| run2 | 0.195 | 0.523 | 1.025 | 34.585*** | 0.000 | 0.044 | 0.205 | 2.112 |
| run3 | 0.215 | 0.509 | 1.045 | 9.547** | 0.005 | 0.049 | 0.225 | 2.295 |
| B vs C | m(C to B) | | | | m(B to C) | | | |
|  | Lower 95% | Peak | Higher 95% | LLRtest | Lower 95% | Peak | Higher 95% | LLRtest |
| run1 | 0.000 | 0.000 | 1.395 | 0.000 | 0 | 0.06 | 0.475 | 1.346 |
| run2 | 0.000 | 0.000 | 1.475 | 0.000 | 0 | 0.043 | 0.465 | 1.546 |
| run3 | 0.000 | 0.000 | 1.505 | 0 | 0 | 0.041 | 0.555 | 2.075 |

* p < 0.05, ** p < 0.01, *** p < 0.001

LLRtest: log likelihood ratio test
