# Supplementary material for: Frequent gene flow blurred taxonomic boundaries of sections in Lilium L. (Liliaceae)
Source: PLoS One. 2017 Aug 25;12(8):e0183209. doi: 10.1371/journal.pone.0183209 (PMC5571923; doi:10.1371/journal.pone.0183209)
Supplement: S5 Table — (DOCX) [file pone.0183209.s005.docx]

**S5 Table.** Summary of indel-sharing events.

| Loci | Position | Length | Taxa with the indel |
| --- | --- | --- | --- |
| EST |  |  |  |
| *LL22* | 458 | 1 | *L.* *taliense* , *L. duchartrei* (sect. *Sinomartagon*) |
| *LL39* | 20 | 12 | *L. tsingtauense*, *L. martagon* (sect. *Martagon*) |
|  | 264 | 3 | *L.* *speciosum* var*. gloriosoides* (sect. *Archelirion*) *in China, L.* *nepalense* (sect. *Sinomartagon*) |
|  | 519 | 13 | *L.* *sulphureum*, *L. sargentiae* (sect. *Leucolirion*), *L. pardalinum*, *L. parryi* (sect. *Pseudolirium*) |

.
