# Supplementary figures and images for: Frequent gene flow blurred taxonomic boundaries of sections in Lilium L. (Liliaceae)
Source: PLoS One. 2017 Aug 25;12(8):e0183209. doi: 10.1371/journal.pone.0183209 (PMC5571923; doi:10.1371/journal.pone.0183209)

## **S1 Appendix. Maximum likelihood phylogenies of 20 EST loci.**

# Lf108

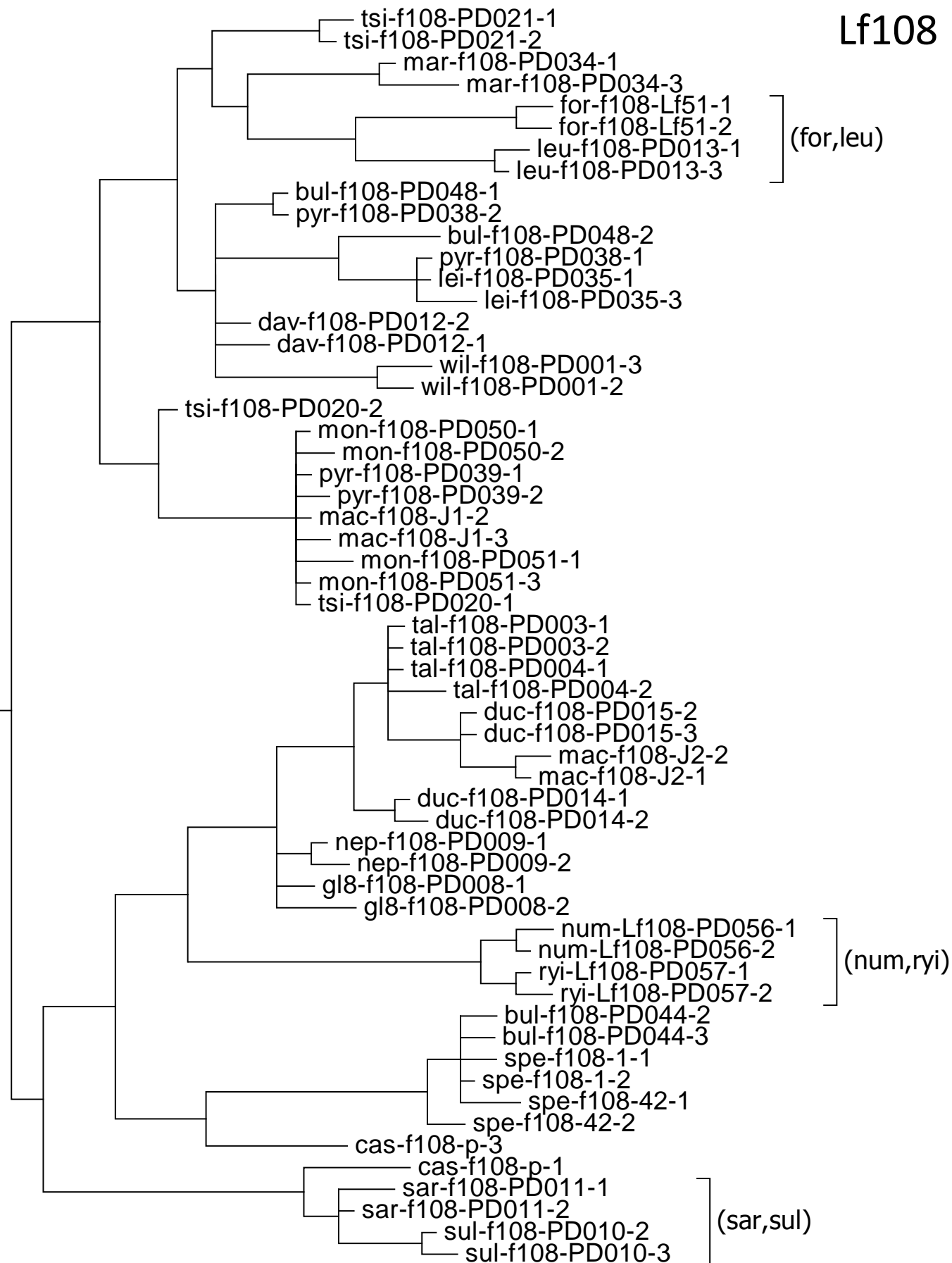

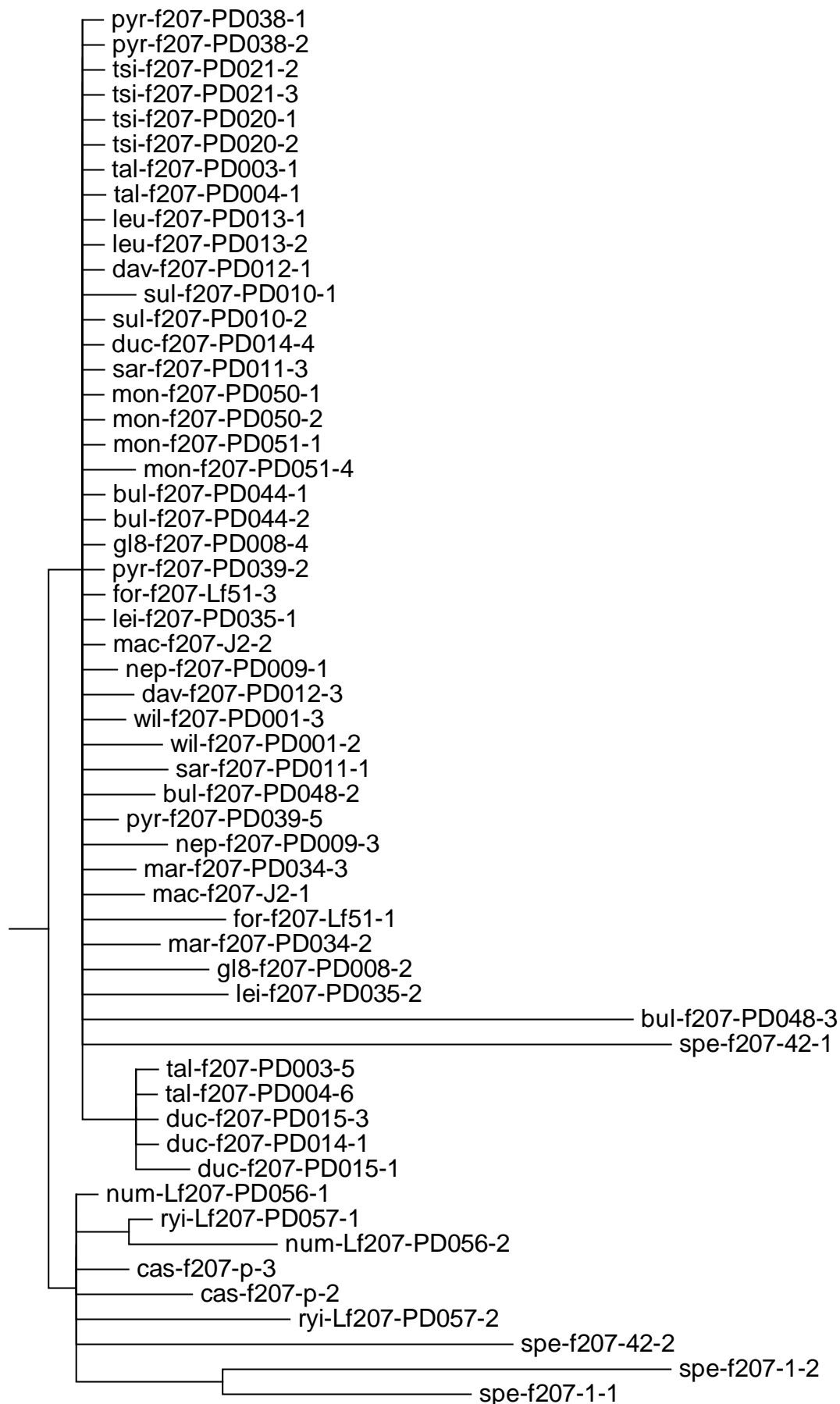

# Lf210

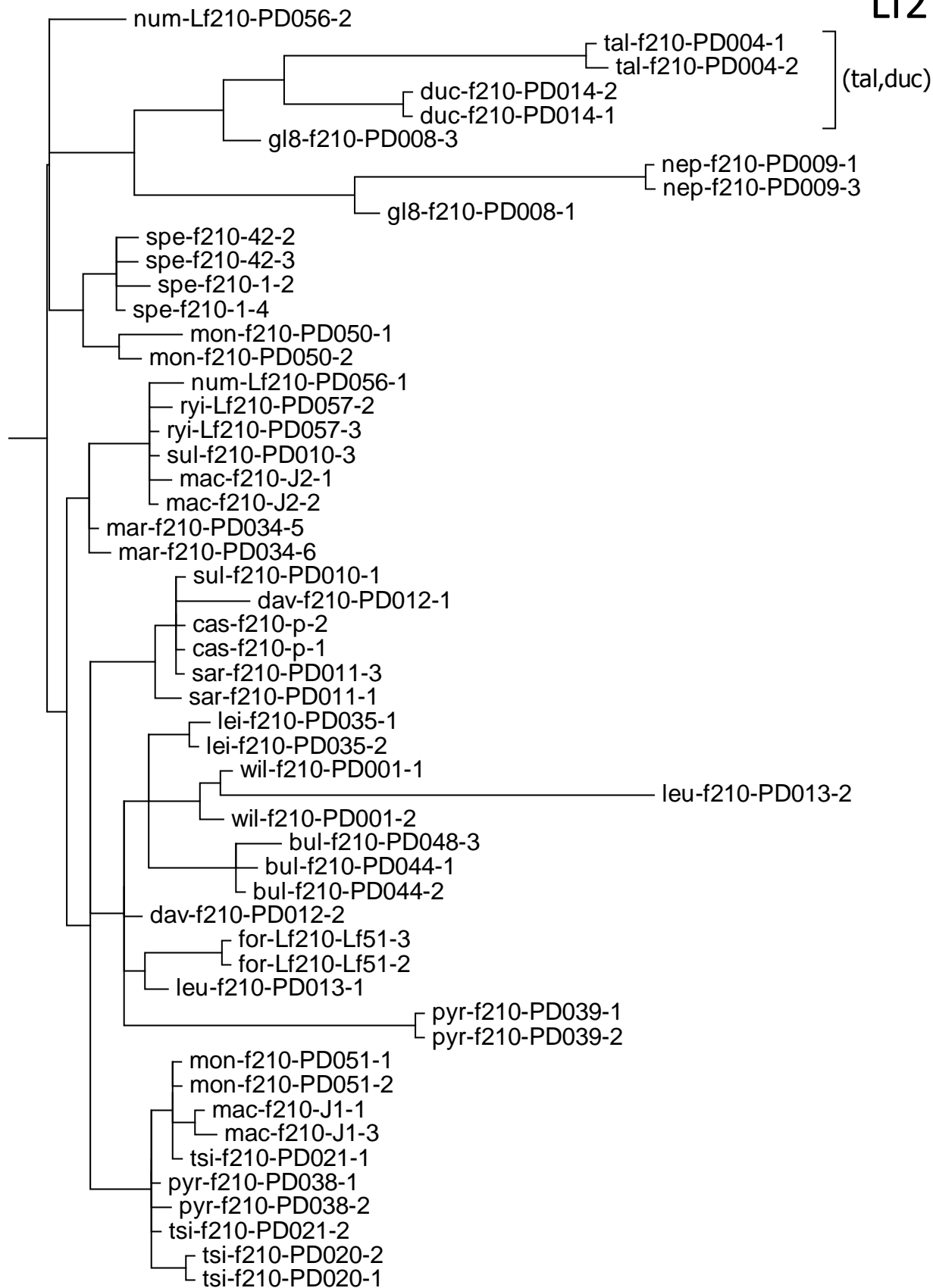

0.01

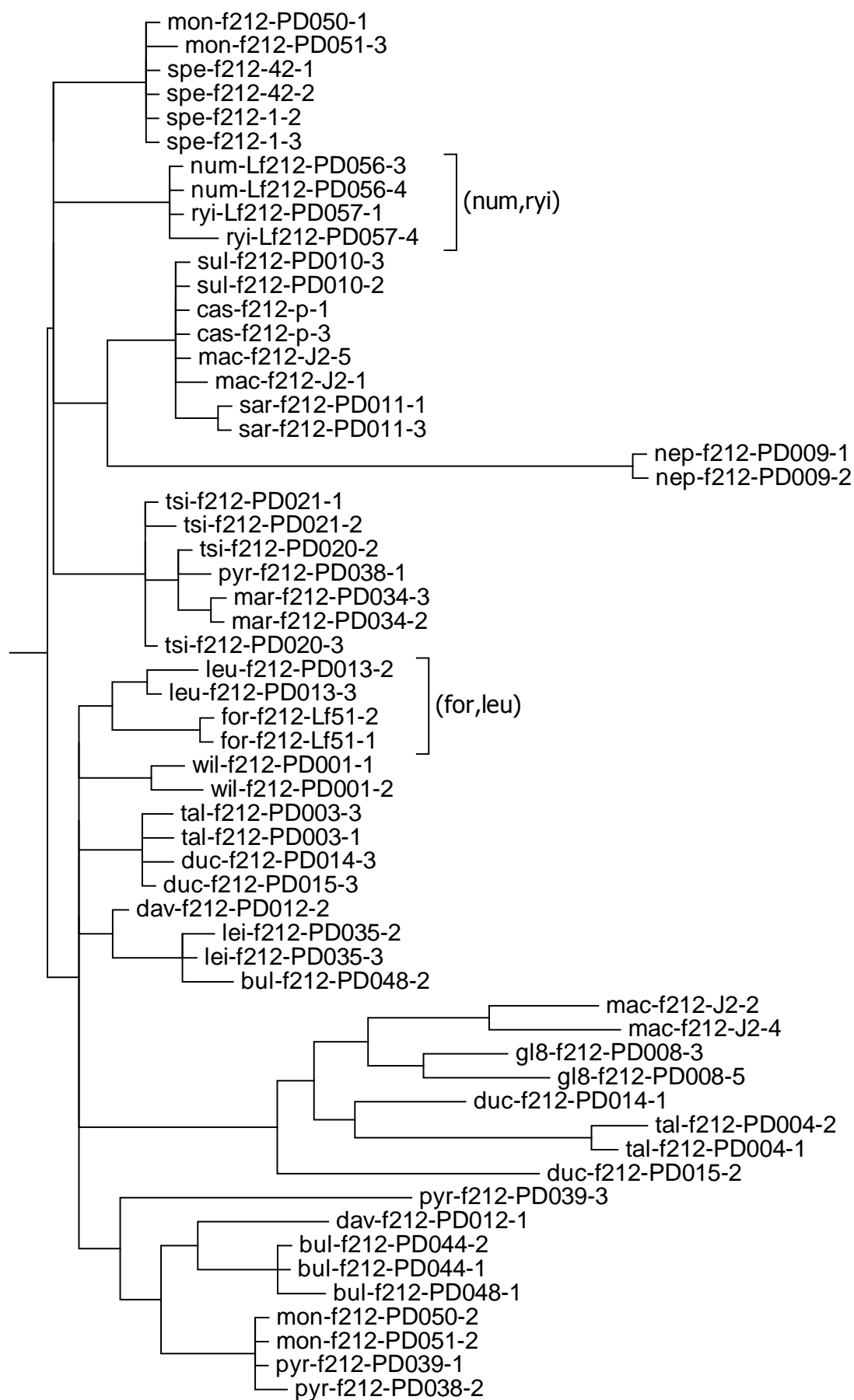

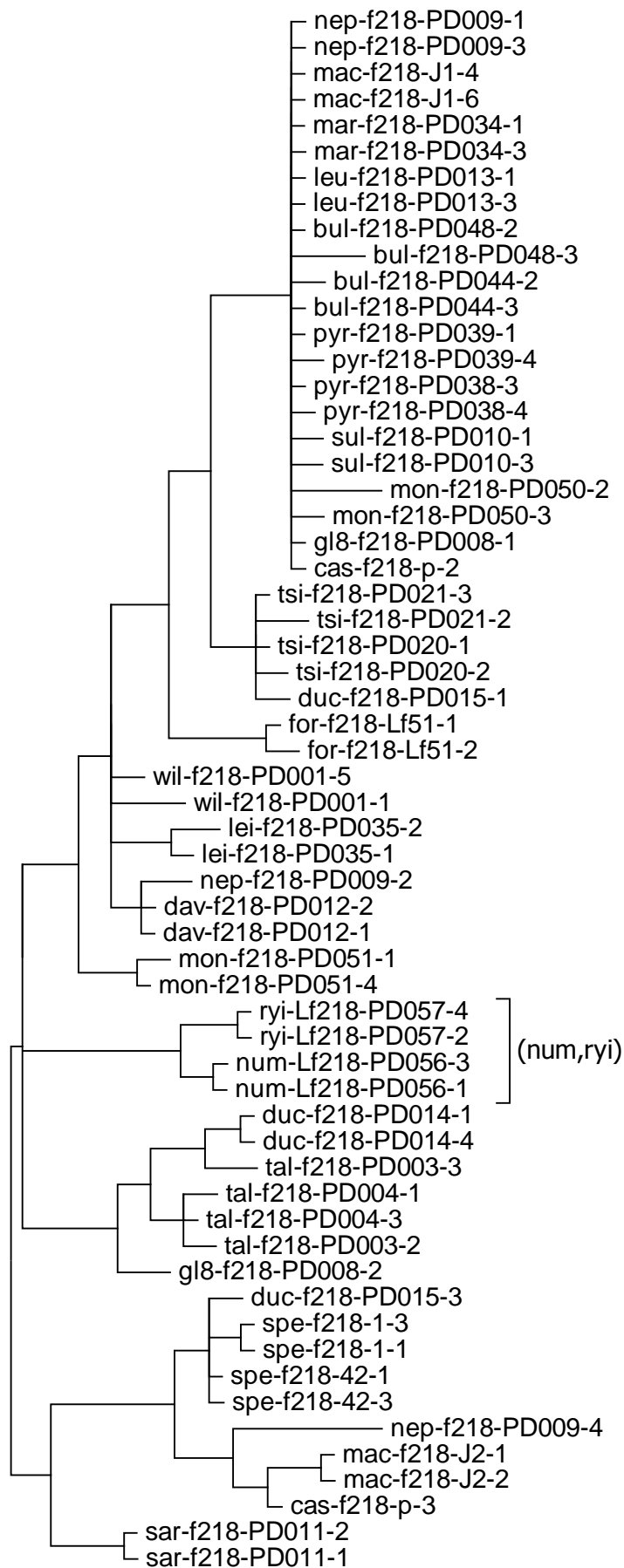

(num,ryi)

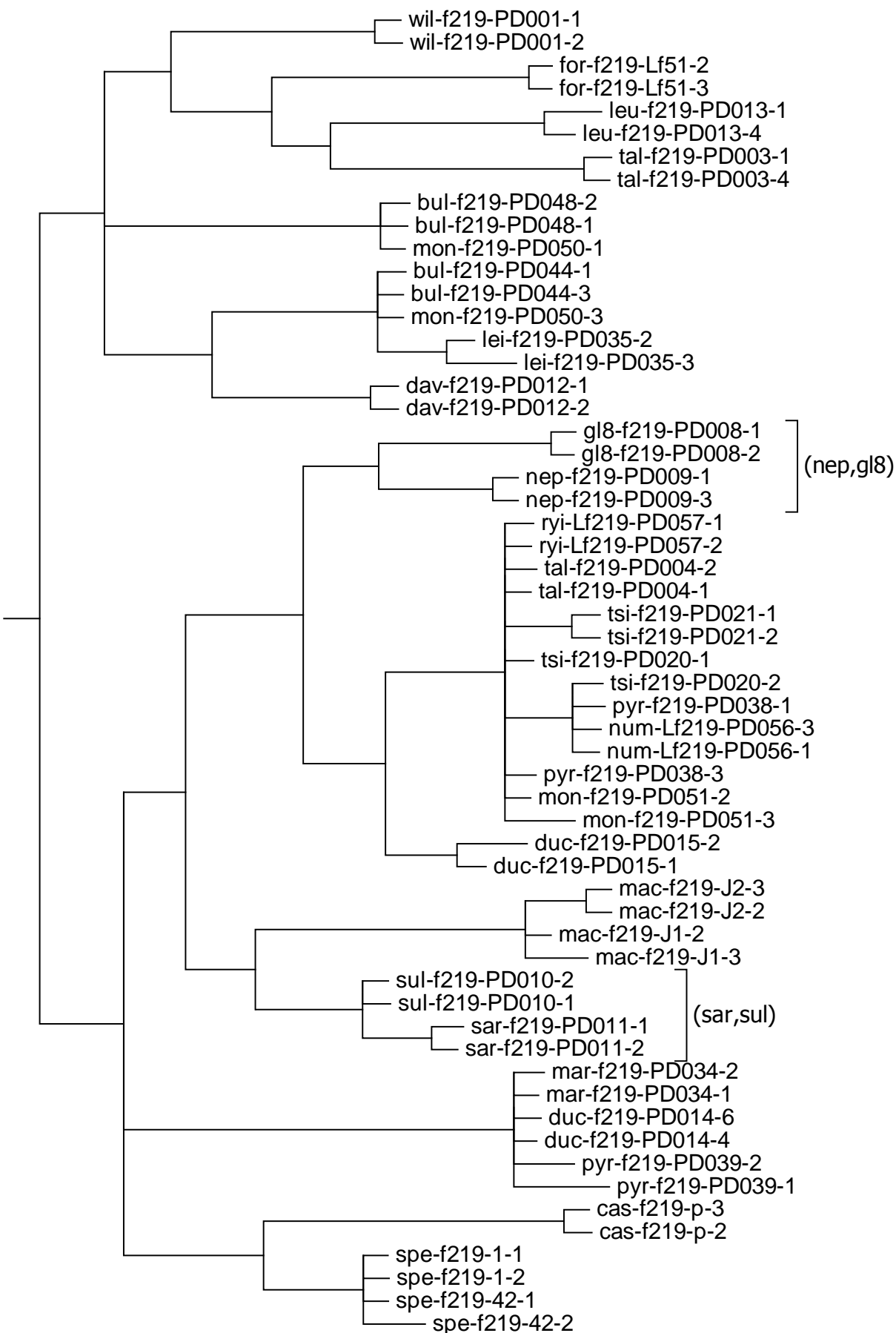

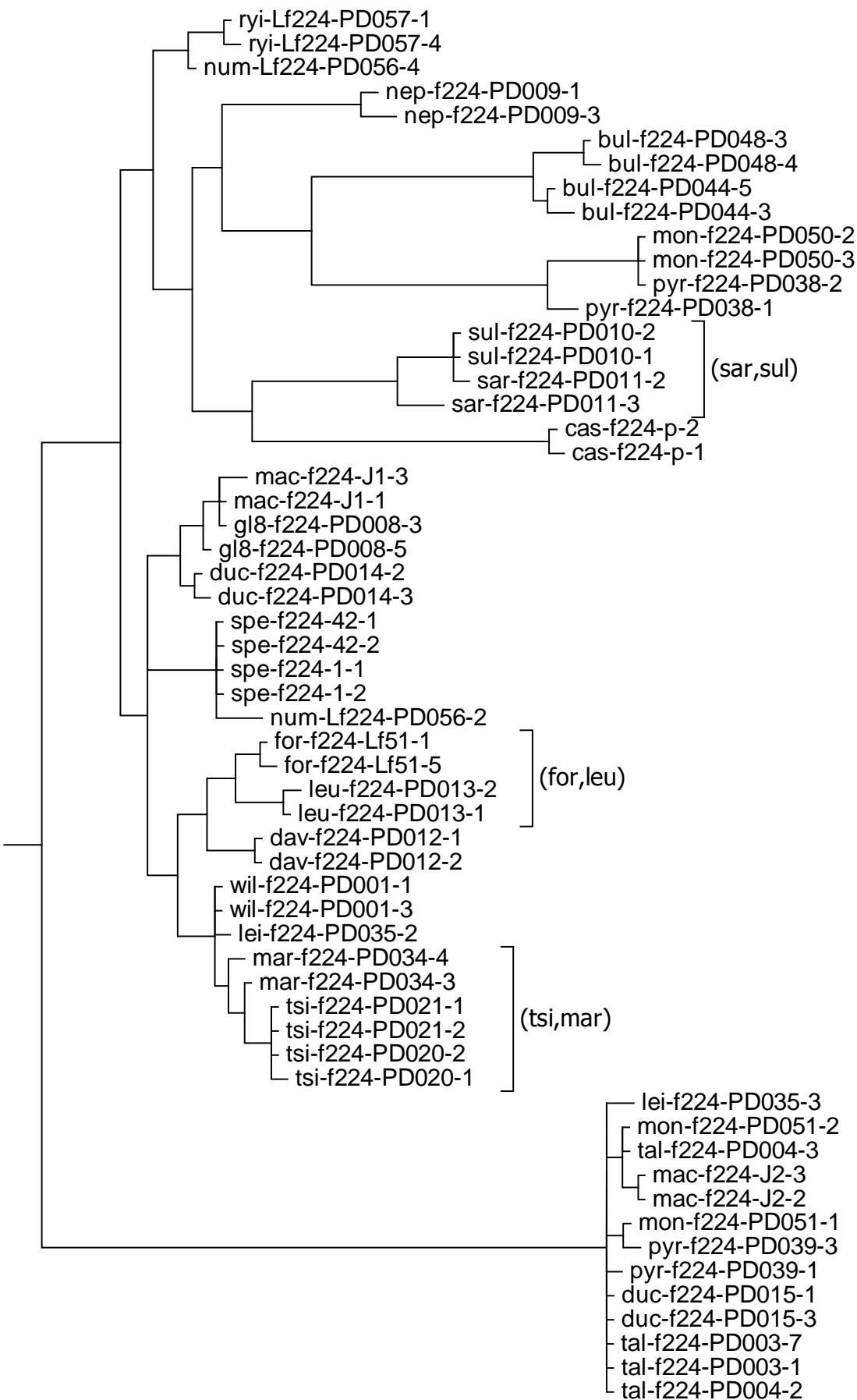

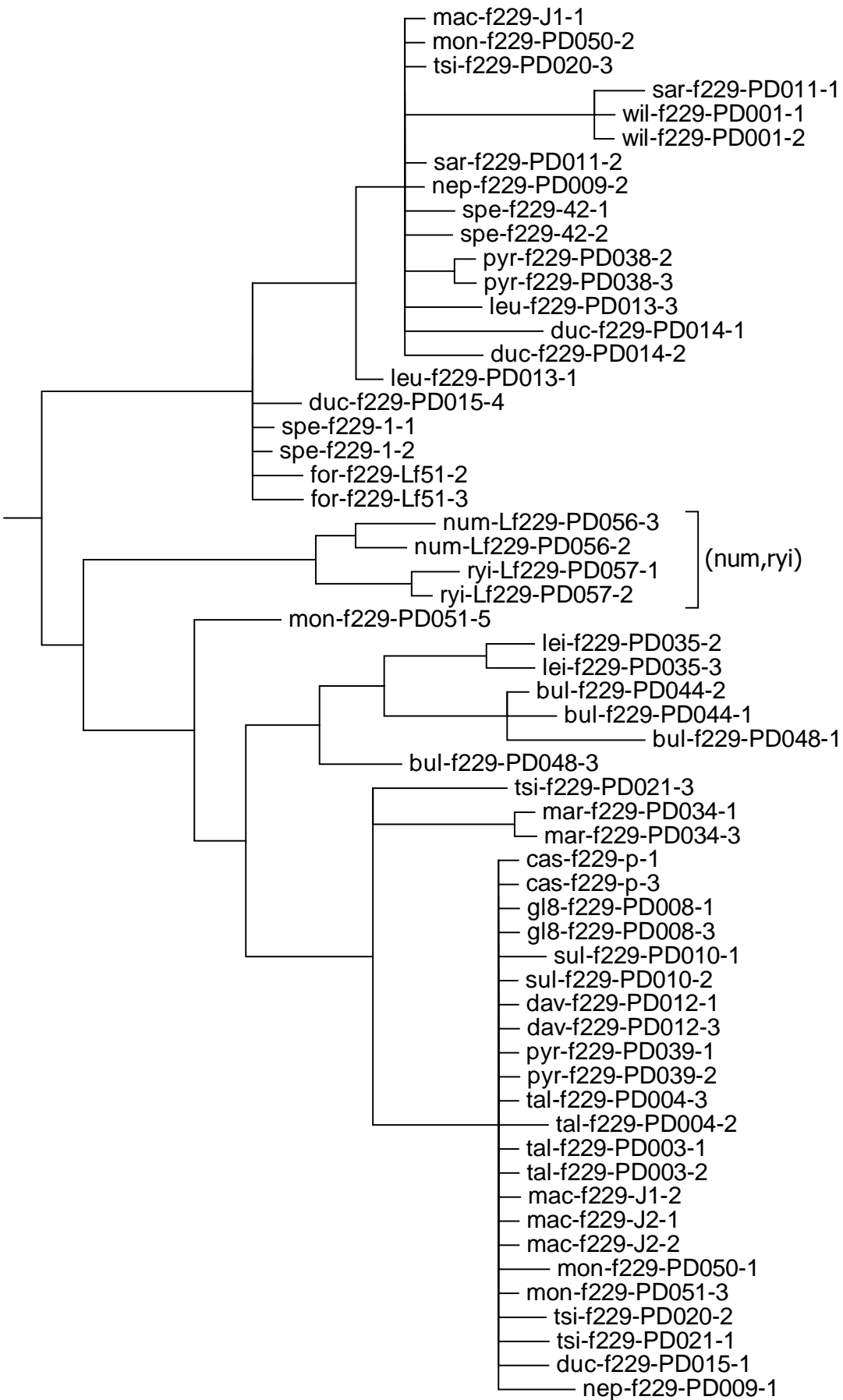

(num,ryi)

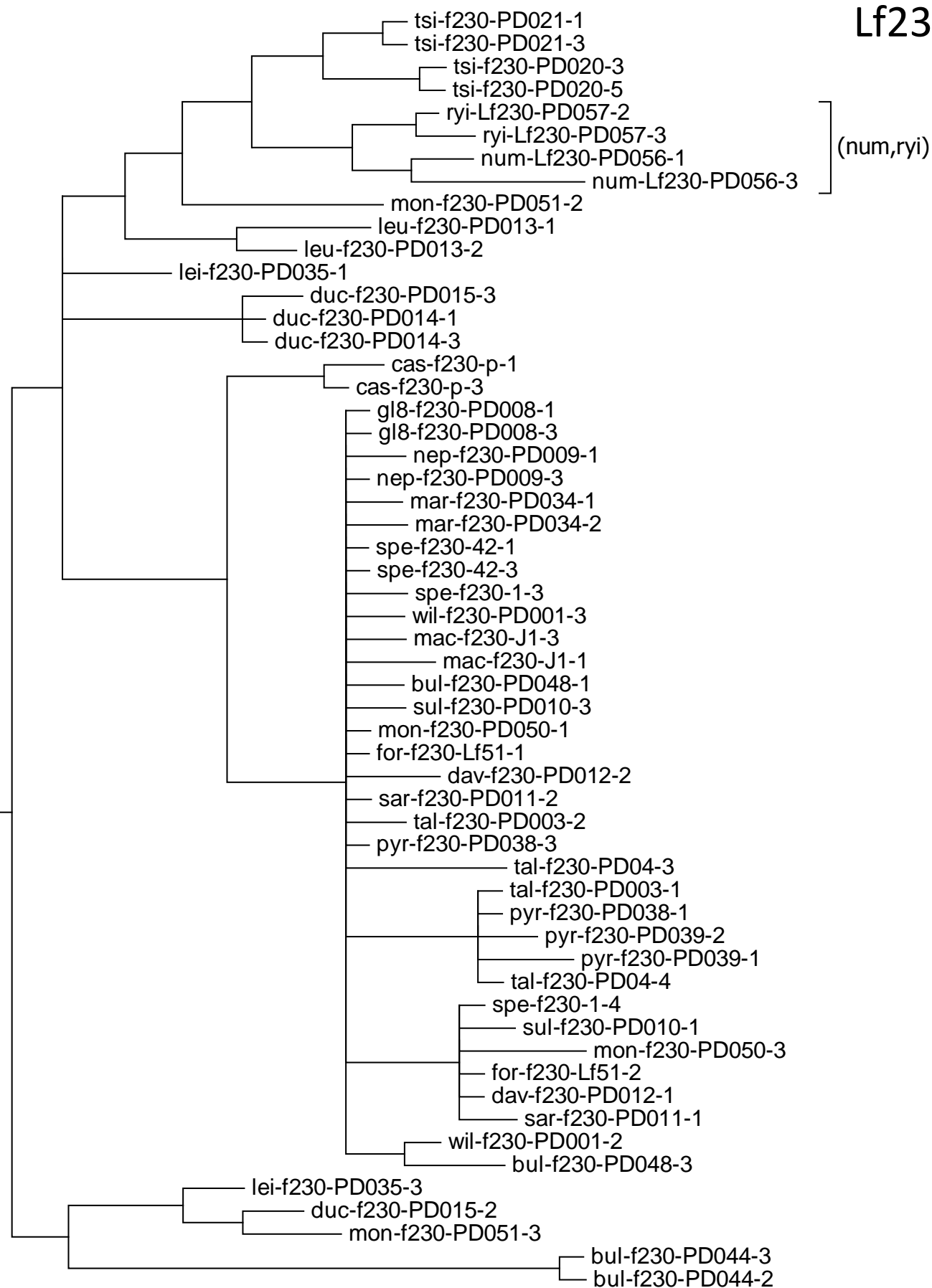

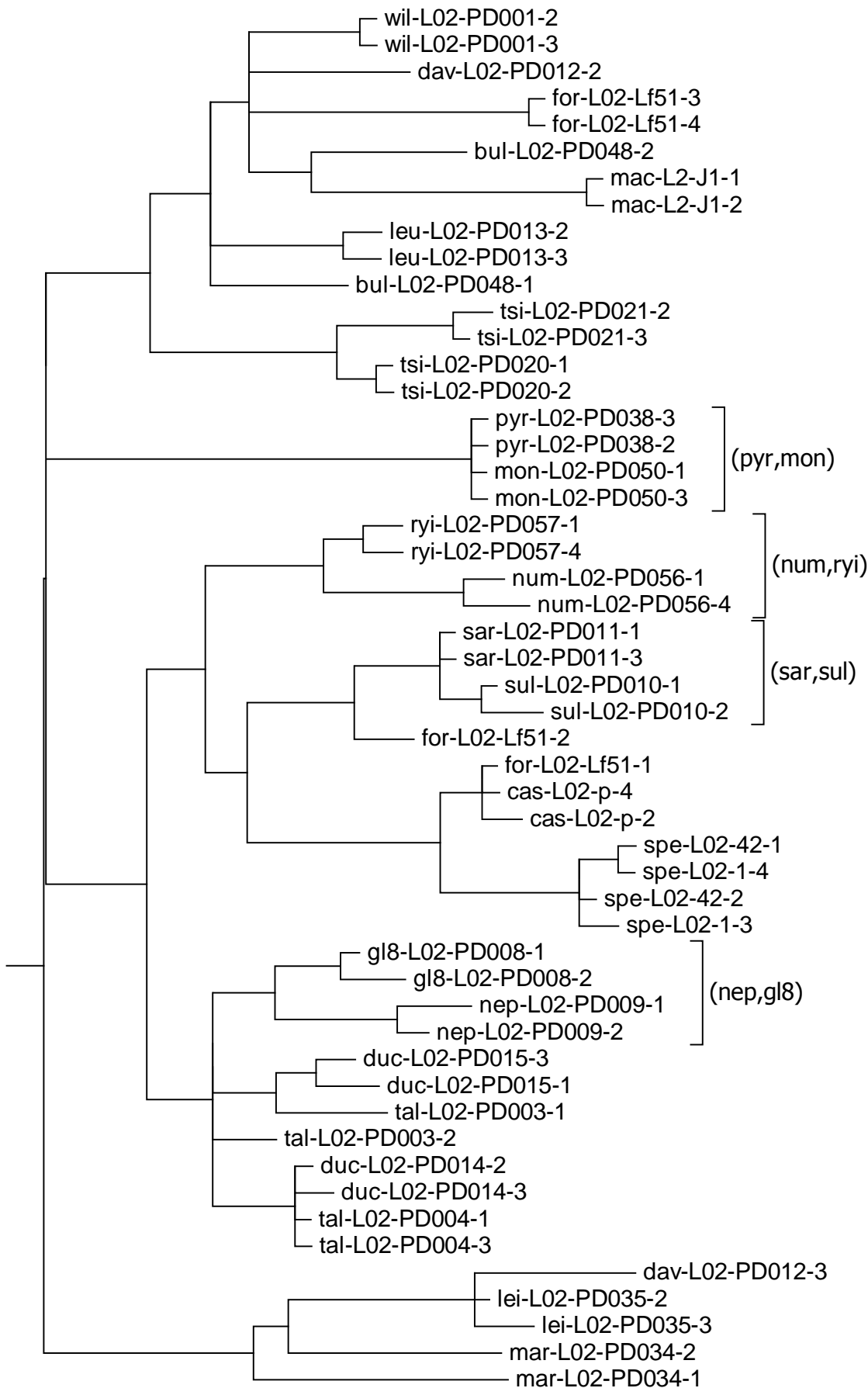

# LL17

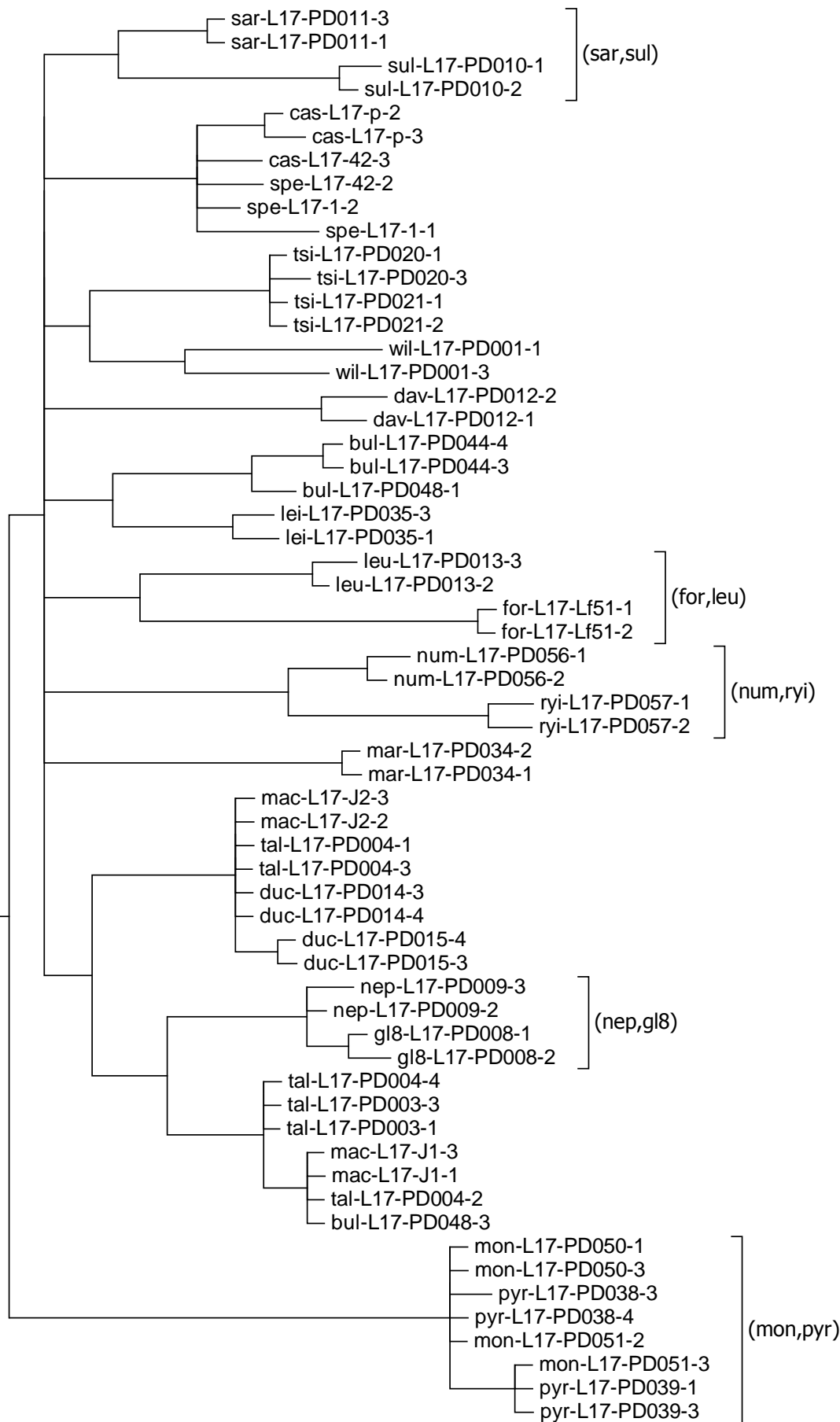

0.005

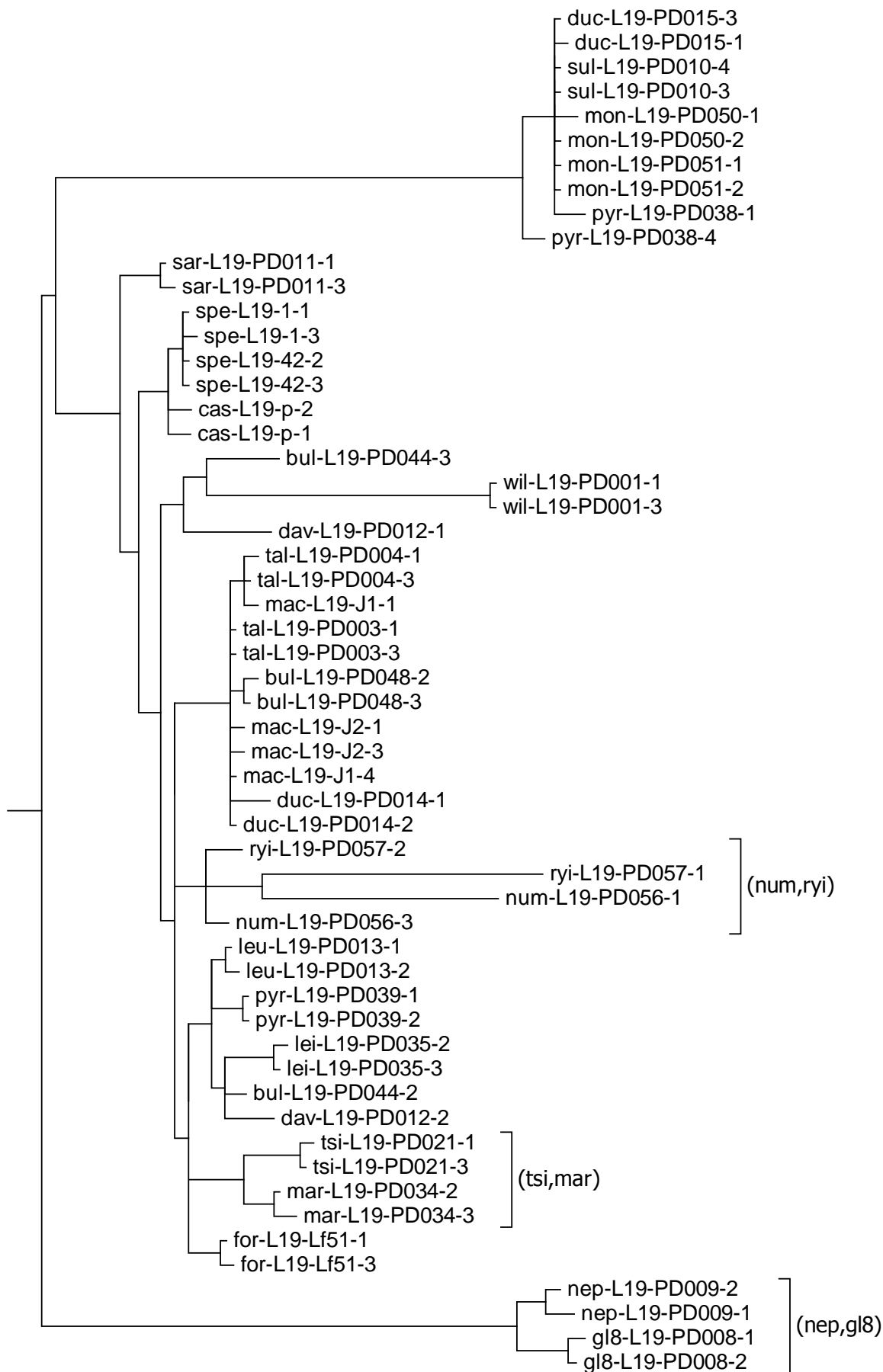

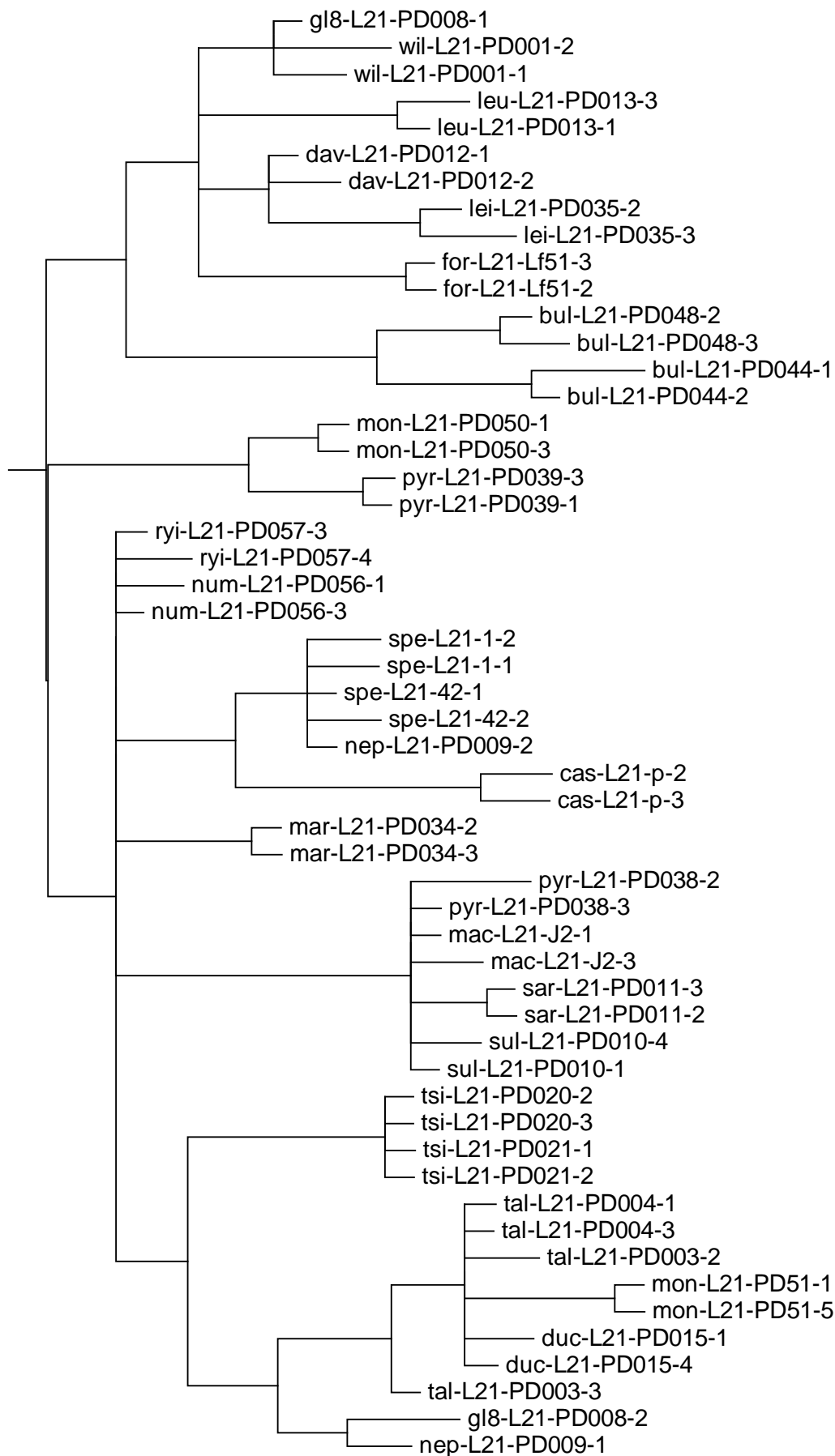

0.002

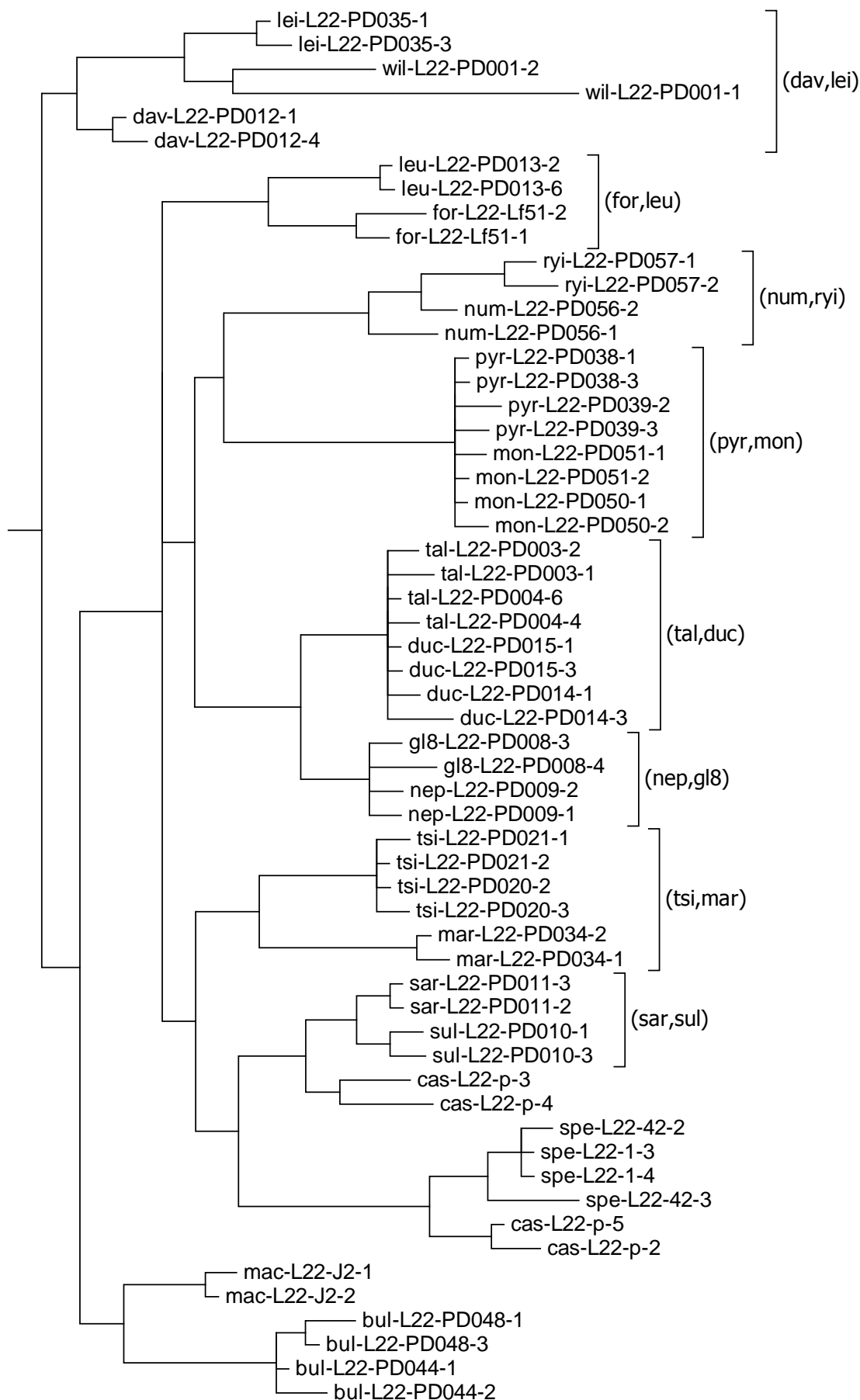

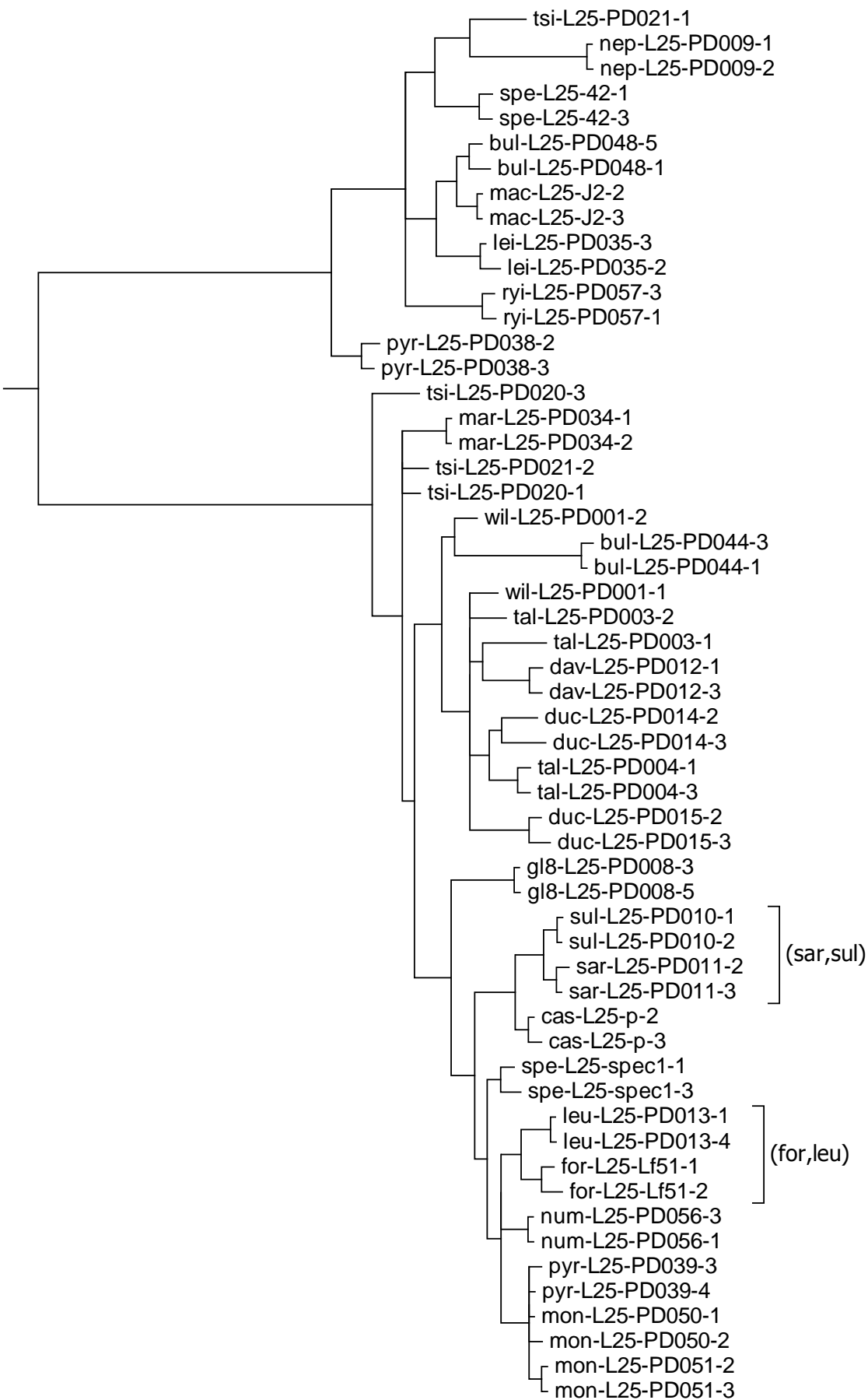

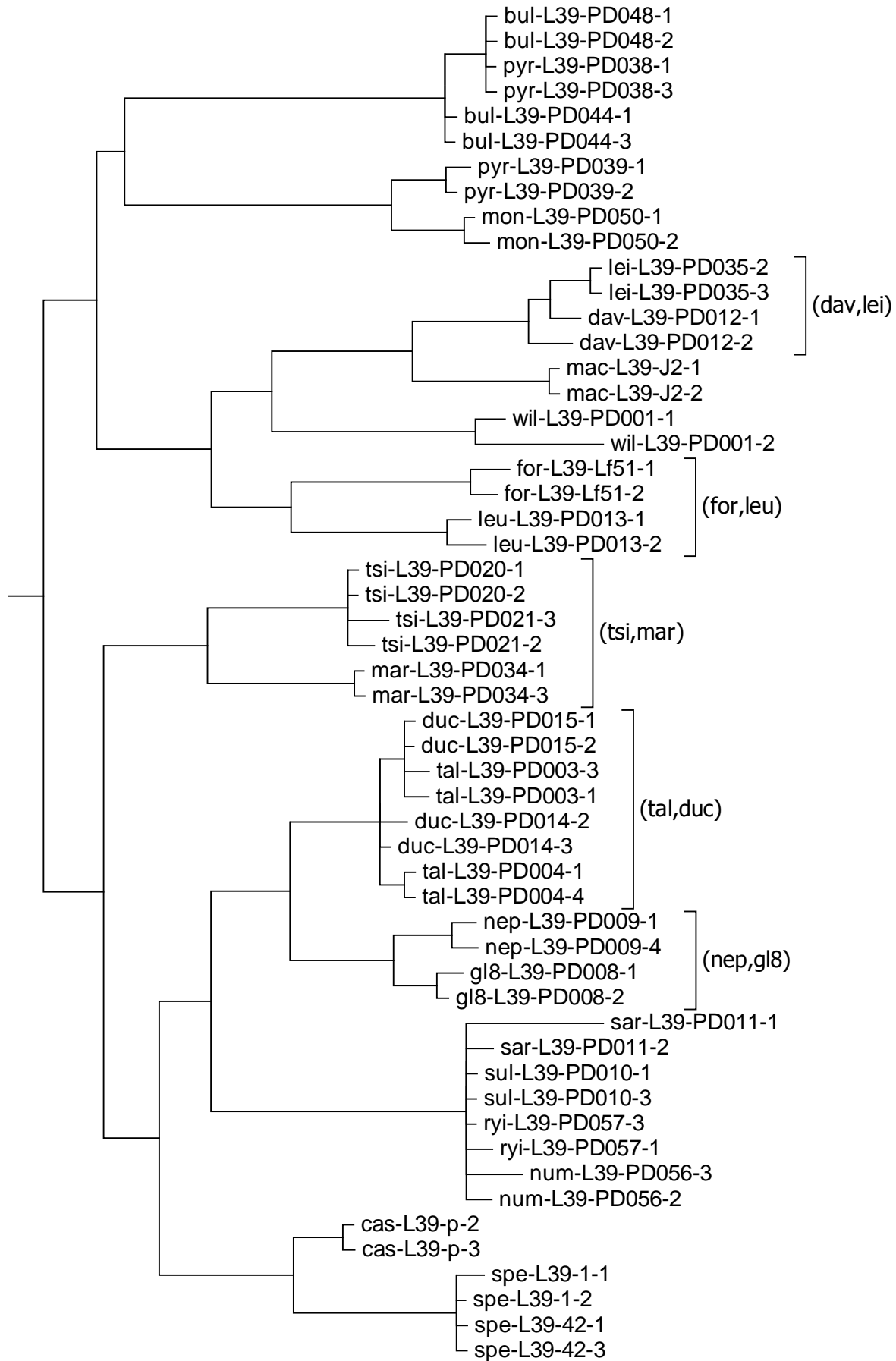

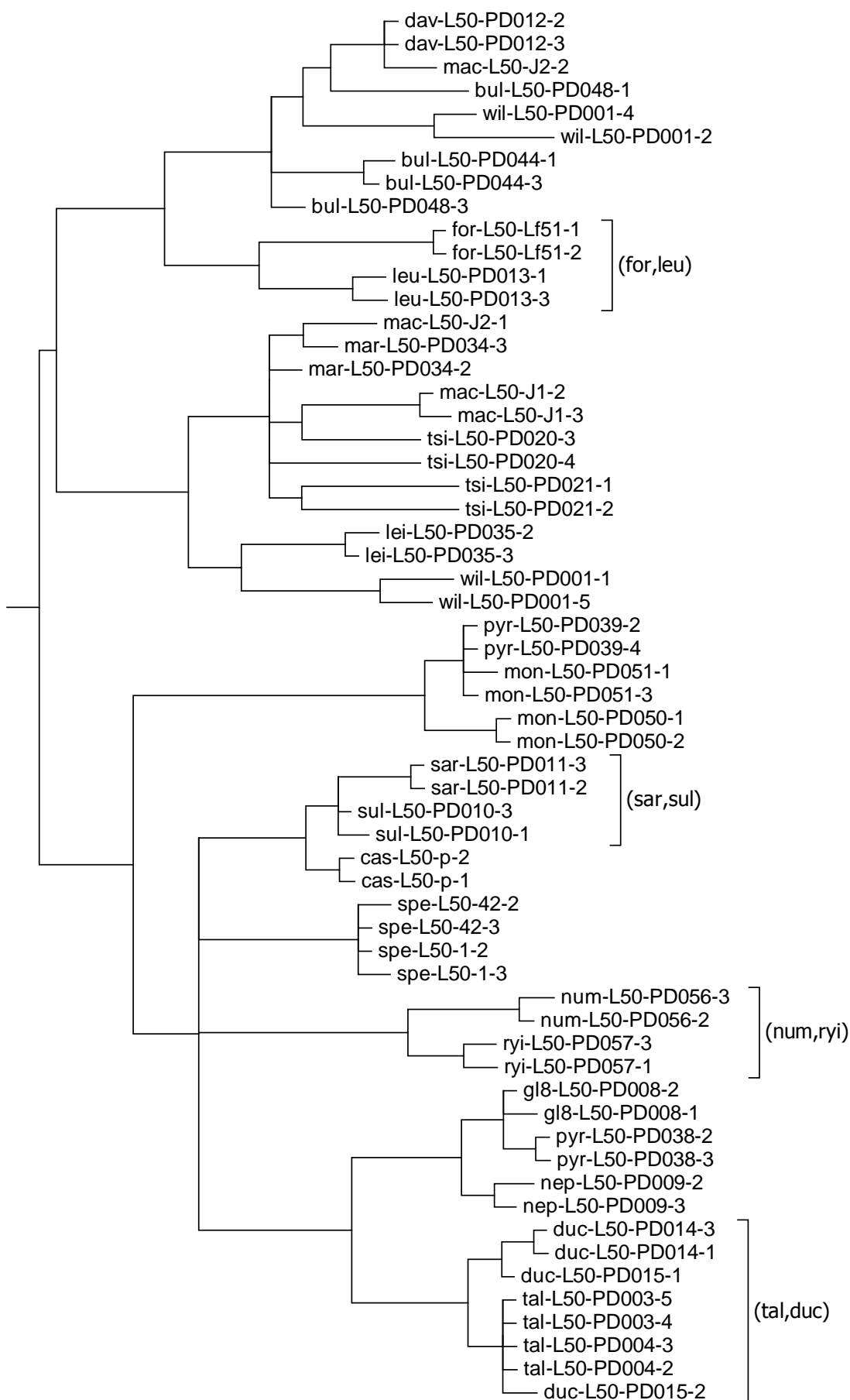

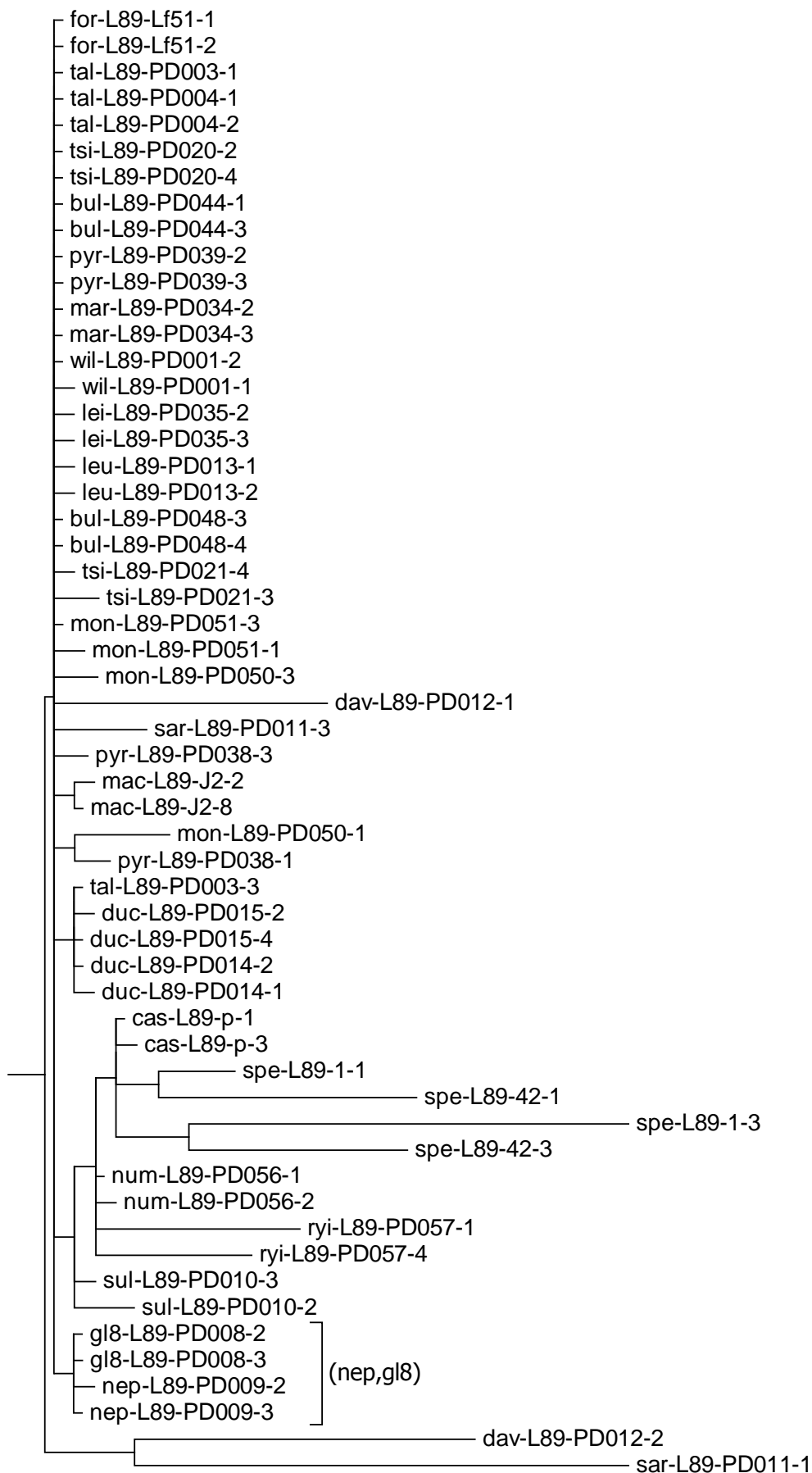

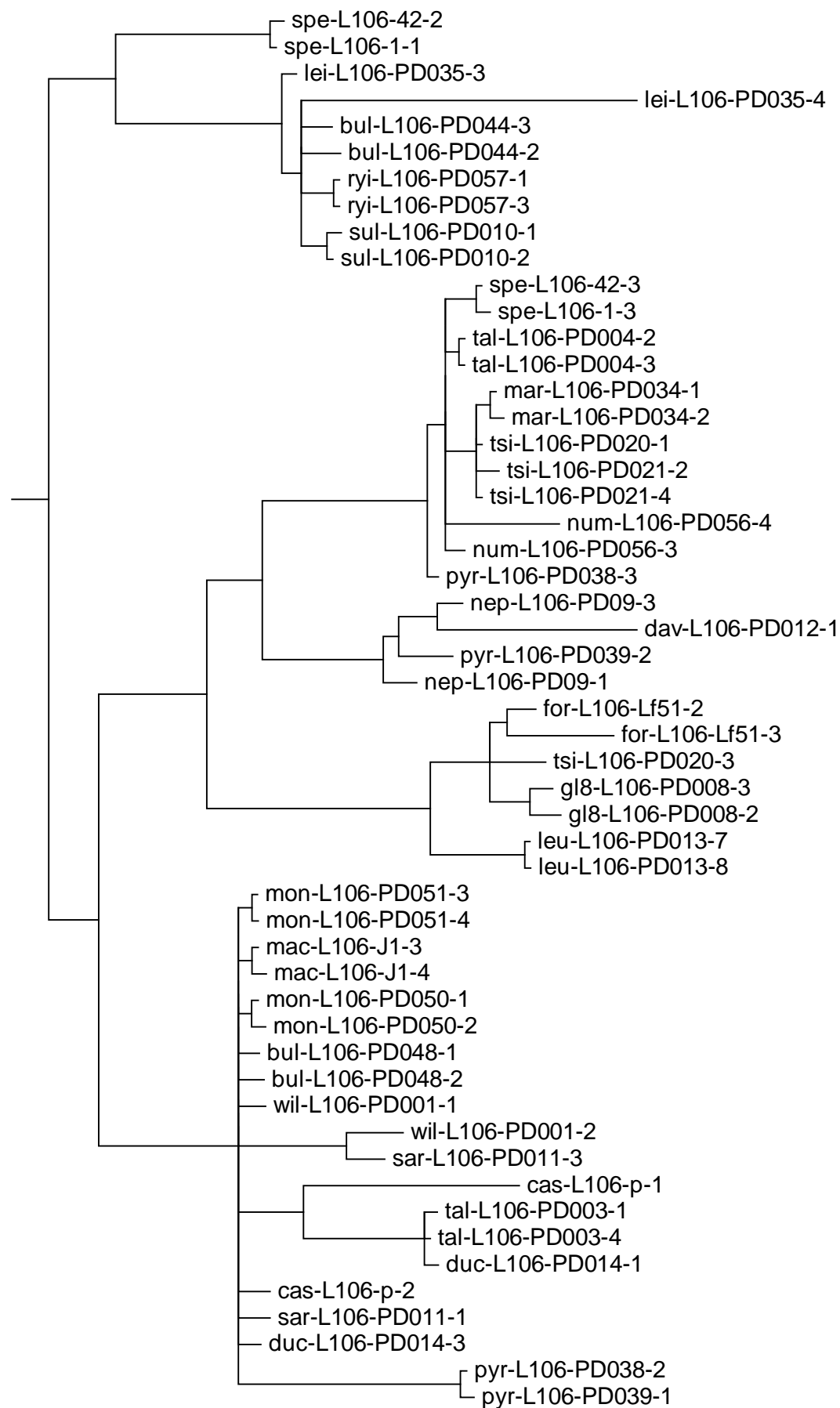

0.05

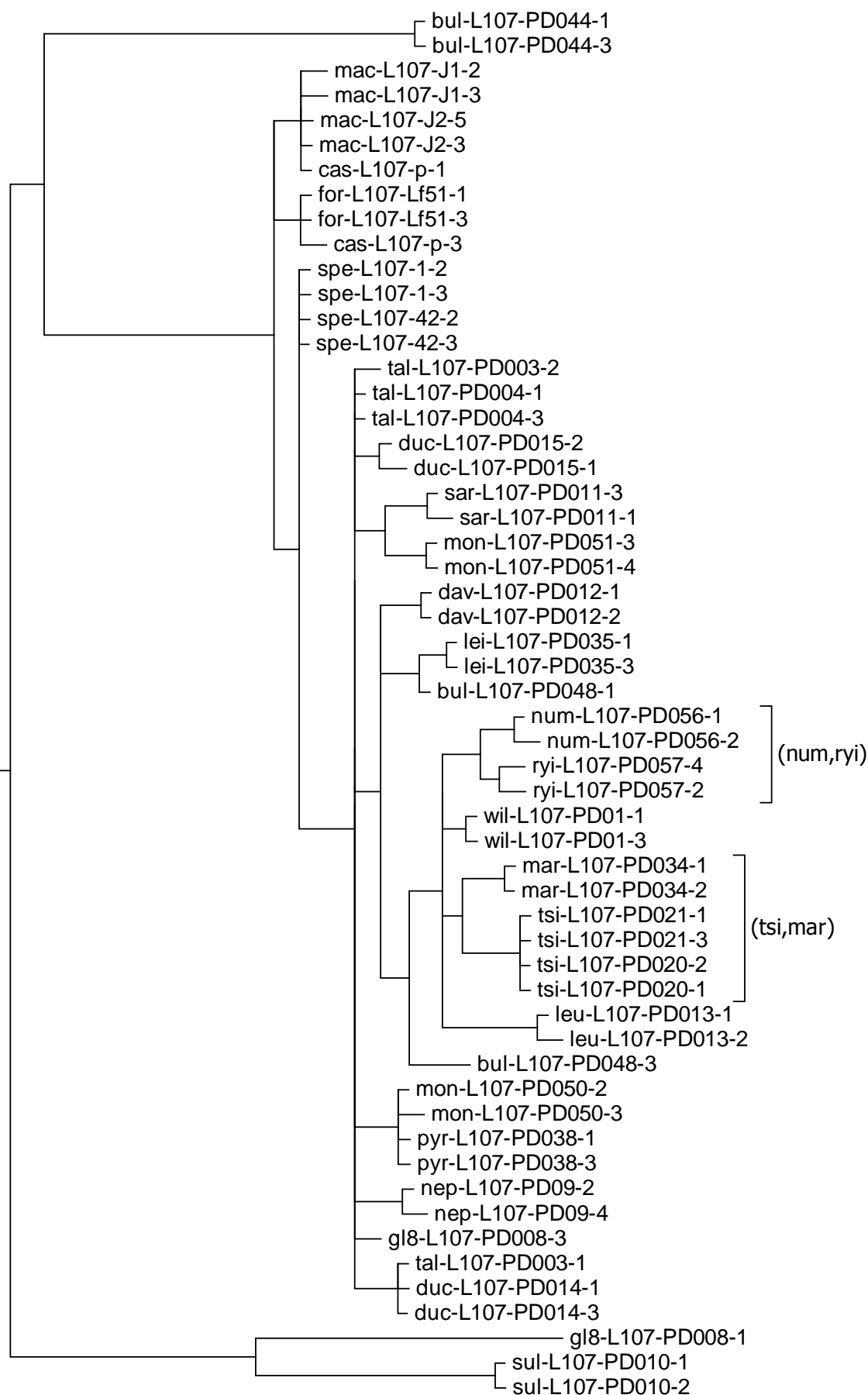

Supplement: S1 Appendix — (PDF) [file pone.0183209.s006.pdf]
